# Integrated Bioinformatics Analysis and In Vitro Evidence Support HSP90AA1 as a Candidate Target of *Camellia petelotii* (Merr.) Sealy in Pulmonary Arterial Hypertension

**DOI:** 10.3390/ijms27083687

**Published:** 2026-04-21

**Authors:** Xinying Chen, Lipeng Zhou, Chenghao Zhu, Zhirong Sun

**Affiliations:** 1 School of Chinese Materia Medica, Beijing University of Chinese Medicine, Beijing 102488, China; 20240935179@bucm.edu.cn (X.C.); 20240941476@bucm.edu.cn (L.Z.); 2 Guangxi Institute of Botany, Chinese Academy of Sciences, Guilin 541006, China

**Keywords:** *Camellia petelotii* (Merr.) Sealy, pulmonary arterial hypertension, bioinformatics, bioactive compounds, HSP90AA1

## Abstract

Pulmonary arterial hypertension (PAH) is a severe and progressive cardiopulmonary disorder with limited treatment options. *Camellia petelotii* (Merr.) Sealy (CP) contains multiple flavonoids and other phytochemicals, but its active compounds and molecular mechanisms in PAH remain unclear. Active compounds of CP were screened by comprehensive literature mining and absorption, distribution, metabolism, and excretion (ADME) evaluation. PAH-related hub targets were identified from transcriptomic data using weighted gene co-expression network analysis (WGCNA), machine learning, and external validation. Functional enrichment, immune infiltration, and single-cell RNA-sequencing analyses were performed to characterize their biological roles and cellular localization. Molecular docking and molecular dynamics simulations assessed compound–target interactions. The effects of CP were further evaluated in hypoxia-induced rat pulmonary artery smooth muscle cells (RPASMCs). Five core bioactive compounds were identified, among which luteolin and quercetin were prioritized for further analysis. *HSP90AA1* and *ROCK2* were screened as hub targets. Bioinformatic analyses suggested that these targets were mainly associated with the “Lipid and atherosclerosis” pathway, metabolic reprogramming, and modulation of the immune microenvironment. Single-cell analysis showed broad expression of *HSP90AA1* and enrichment of *ROCK2* in fibroblasts and endothelial cells. Molecular docking and molecular dynamics simulations supported stable binding of luteolin to HSP90AA1. In vitro, CP extract inhibited hypoxia-induced hyperproliferation of RPASMCs and reduced HSP90AA1 protein expression. HSP90AA1 may represent a candidate molecular mediator of CP in PAH, and CP inhibited hypoxia-induced RPASMC proliferation in association with downregulation of HSP90AA1.

## 1. Introduction

Pulmonary hypertension is a heterogeneous group of cardiopulmonary disorders, among which pulmonary arterial hypertension (PAH) is a severe subtype characterized by progressive pulmonary vascular remodeling, increased pulmonary vascular resistance, and eventual right ventricular failure [1,2,3]. Current therapies primarily target four key endothelial pathways: endothelin-1, nitric oxide, prostacyclin, and bone morphogenetic protein/activin signaling. Despite substantial progress in treatment over recent decades, PAH remains incurable and is still associated with poor prognosis, especially in intermediate- and high-risk patients [4,5]. Consequently, there remains an urgent need to develop novel therapeutic agents for PAH with high efficacy and safety.

*Camellia petelotii* (Merr.) Sealy (CP), commonly known as “Jinhuacha”, is a traditional medicinal and edible plant native to southwestern China, particularly Guangxi Province, where it has long been used among Zhuang ethnic communities. According to the Guangxi Traditional Chinese Medicinal Materials Standard: Second Volume (1996) [6], CP has traditionally been used in the management of hypertension. Phytochemical studies have shown that CP contains tea polyphenols, polysaccharides, flavonoids, saponins, amino acids, and trace elements. Among these constituents, flavonoids and tea polyphenols are considered major bioactive components with antioxidant, anti-inflammatory, and cardiovascular-protective properties [7,8,9,10,11]. Previous studies have also suggested that CP extracts can improve lipid metabolism, reduce blood pressure, and attenuate oxidative stress in experimental models [12,13,14]. These pharmacological properties are closely related to key pathological processes involved in PAH, including vascular remodeling, oxidative injury, inflammation, and abnormal proliferation of pulmonary artery smooth muscle cells. Therefore, although CP has traditionally been applied in systemic hypertension, we hypothesized that CP may also exert therapeutic effects against PAH by acting on specific molecular targets in disease-relevant vascular cells.

The study of natural medicines in PAH is inherently challenging. Their effects may be related to multiple compounds, targets, pathways, and cell types. Conventional network pharmacology and transcriptomic approaches are useful for target identification. However, these methods cannot fully resolve the cellular context within the pulmonary microenvironment. PAH involves multiple cell types in the vascular wall and surrounding tissue. Accordingly, a more integrated analytical strategy is needed to connect candidate compounds with disease-relevant targets and their cellular localization.

**Figure 1:**
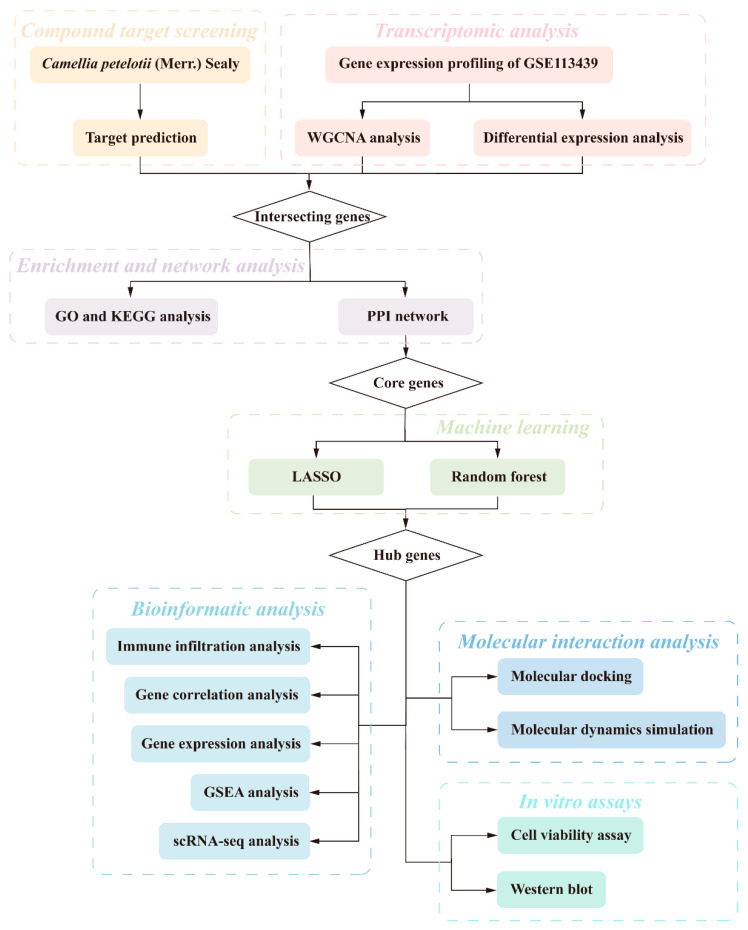
Overview of the methodological workflow. WGCNA, weighted gene co-expression network analysis; GO, Gene Ontology; KEGG, Kyoto Encyclopedia of Genes and Genomes; PPI, protein–protein interaction; LASSO, least absolute shrinkage and selection operator; GSEA, gene set enrichment analysis; scRNA-seq, single-cell RNA sequencing.

In this study, we combined target prediction, cell-specific localization, and in vitro experiments to identify a candidate target of CP in PAH, marking the first application of this integrated strategy in this context. This approach allowed the predicted targets to be evaluated not only at the molecular level but also in relation to their distribution in relevant vascular cell populations, thereby providing a more comprehensive basis for further investigation. First, network pharmacology and machine learning algorithms were used to predict hub targets. Subsequently, functional enrichment and immune infiltration analyses were performed to investigate the underlying biological pathways and the associations between targets and the immune microenvironment. Next, scRNA-seq analysis was performed to determine the specific cellular localization of the predicted targets, followed by molecular docking and molecular dynamics simulations to assess the physical binding stability of active constituents to these targets. Finally, a hypoxia-induced rat pulmonary artery smooth muscle cell (RPASMC) model was established to evaluate the biological effects of CP extract and to verify the regulation of the predicted hub target in vitro. Our methodological workflow is shown in Figure 1.

## 2. Results

### 2.1. Screening of Bioactive Compounds and Potential Targets of CP

**Figure 2:**
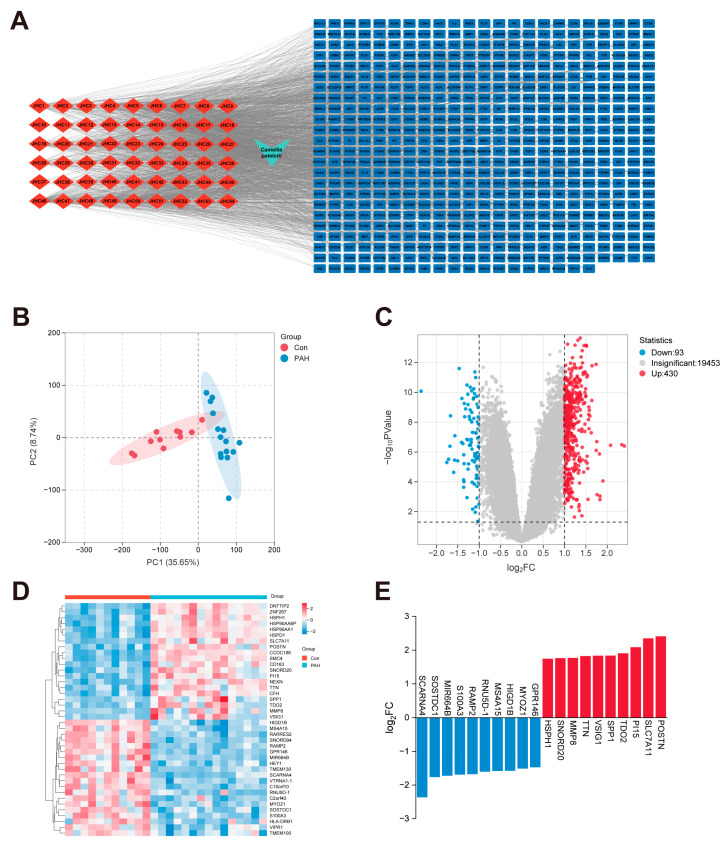
Targets of *Camellia petelotii* (Merr.) Sealy active components and differential expression genes in PAH. (**A**) Active compound-target network of CP. (**B**) Principal component analysis (PCA) plot of the GSE113439 dataset. (**C**) Volcano plot of differentially expressed genes (DEGs). (**D**) Heatmap of top 20 DEGs. (**E**) Log_2_ fold change (Log_2_FC) values of the top 10 up- and down-regulated genes.

According to the published literature summarized in Supplementary Table S1, 181 plant compounds of CP were collected. The corresponding literature sources for compound collection are listed separately within Supplementary Table S1. After evaluation using the SwissADME platform, 54 compounds were retained as candidate bioactive constituents (JHC1–JHC54). Detailed information was given in Table 1. These compounds were then mapped to 548 potential targets using the Swiss Target Prediction database. A compound–target network was constructed using Cytoscape 3.10.0, and 5 candidate compounds were prioritized based on degree values, including 3-trans-5-cis-pseudoionone, blumenol C, luteolin, quercetin and kaempferol (Figure 2A).

### 2.2. Screening of PAH-Related DEGs

We selected the GSE113439 dataset from the GEO database. PCA of the normalized transcriptome profiles revealed that the samples had less intersection and better aggregation, demonstrating good representativeness of the groups (Figure 2B). Differential gene expression analysis identified a total of 523 DEGs, comprising 430 up-regulated genes and 93 down-regulated genes. The distribution of the differential genes was displayed in a volcano plot (Figure 2C). The top 20 DEGs were plotted in a clustered heat map (Figure 2D). The histogram of the top 10 DEGs was shown in Figure 2E.

### 2.3. Screening of WGCNA Module Genes

**Figure 3:**
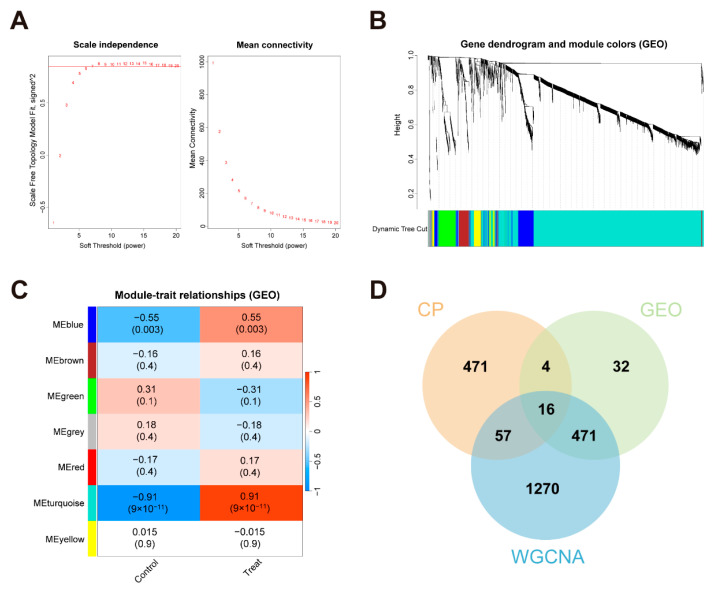
WGCNA analysis and screening of intersecting genes. (**A**) Soft threshold β = 7 and scale-free topological fit index (R^2^). (**B**) Merged clustering dendrogram of WGCNA genes, with different colors indicating different modules identified by dynamic tree cut. (**C**) Heatmap of the module–trait relationships. (**D**) Venn diagram of intersecting targets between CP active components and PAH-related targets.

In order to construct a scale-free co-expression network, we selected the optimal value for the soft threshold of the adjacency matrix based on the scale-free topology standard (R^2^ = 0.85). When the power parameter was set to 7, the correlation coefficients and the average connectivity could reach their highest levels at the same time (Figure 3A).

The dynamic tree-cutting algorithm was utilized to identify distinct modules, and a dissimilarity threshold of 0.25 was used for similar module clustering. After merging similar modules, all the PAH-related genes were classified into 7 modules, as presented in the gene clustering tree diagram (Figure 3B). The heatmap was generated based on correlation coefficients and *p*-value module characteristic genes with the traits (Figure 3C). The result indicated that the turquoise module, containing 1814 genes, had the most significant *p*-value. Through intersection calculation of target genes of CP, DEGs and WGCNA module genes, 16 intersecting genes were obtained for subsequent screening of core genes (Figure 3D) and all related genes are listed in Supplementary Table S2.

### 2.4. Construction of PPI Network

**Figure 4:**
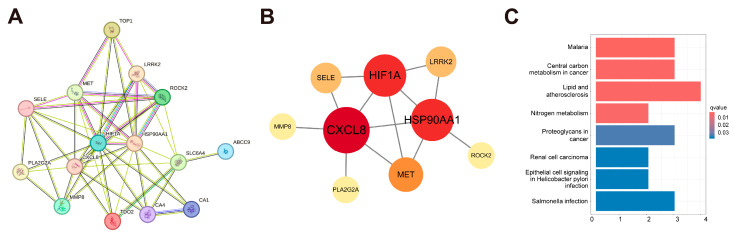
PPI network and KEGG enrichment analysis. (**A**) PPI network. (**B**) Map of core targets. (**C**) KEGG enrichment analysis.

We imported 16 overlapping targets into the STRING database to construct the PPI network and further analyzed it in Cytoscape 3.10.0. After removal of isolated nodes without interactions, nine connected targets were retained and defined as core targets for subsequent analysis, including *CXCL8*, *HSP90AA1*, *HIF1A*, *MET*, *SELE*, *LRRK2*, *MMP8*, *PLA2G2A*, and *ROCK2*, ranked in descending order by degree value (Figure 4A,B). In Figure 4B, the darker red color and the larger circle represent the larger degree value.

### 2.5. GO and KEGG Enrichment Analysis

GO enrichment analysis identified a total of 814 enriched terms, including 635 terms linked to biological processes (BP), 57 terms related to cellular component (CC) and 122 terms related to molecular function (MF) [15]. The top 10 enriched terms were selected to be visualized in bubble plots separately (Supplementary Figure S1). BP categories are significantly enriched in the regulation of G protein-coupled receptors. CC terms highlighted the membrane raft and membrane microdomain. The MF processes are primarily mediated through transmembrane transporter binding, GTPase binding, histone H2AX kinase activity, and histone H3 kinase activity. KEGG pathway enrichment analysis screened 8 signaling pathways. As shown in Figure 4C, the intersecting genes were mainly enriched in the “lipid and atherosclerosis” pathway, which showed the highest gene ratio and the lowest q value among the enriched pathways, suggesting that it may be relevant to PAH-associated molecular alterations.

### 2.6. Machine Learning-Based Identification of Hub Genes

**Figure 5:**
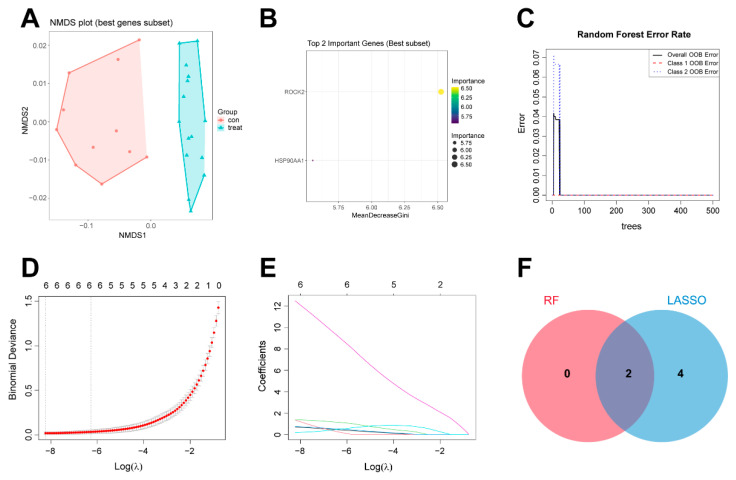
Hub gene identification based on machine learning algorithms. (**A**) Non-metric multidimensional scaling (NMDS) analysis of the RF method. (**B**) The importance evaluation of the random forest (RF) method. (**C**) The error rate curves of the RF method. (**D**) The regularization diagrams of LASSO analysis. (**E**) The coefficient diagrams of LASSO analysis. Different colored lines indicate different genes: magenta for *ROCK2*, green for *HIF1A*, red for *HSP90AA1*, blue for *PLA2G2A*, black for *CXCL8*, and cyan for *MET*. (**F**) Venn diagram of intersecting targets between RF and LASSO analysis.

Random forest algorithm filtered 2 genes, comprising *HSP90AA1* and *ROCK2* (Figure 5A–C). LASSO analysis identified 6 candidate genes: *CXCL8*, *HSP90AA1*, *HIF1A*, *PLA2G2A*, *MET*, *ROCK2* (Figure 5D,E). Intersection of both analyses pinpointed *HSP90AA1* and *ROCK2* as hub genes most strongly associated with the predicted effects of CP in PAH (Figure 5F).

### 2.7. Immune Cell Infiltration and Correlation with Hub Genes

**Figure 6:**
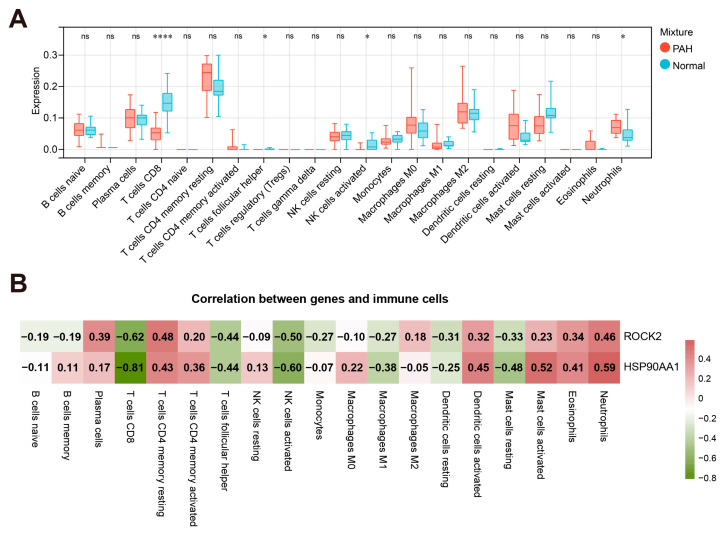
Immune infiltration analysis of hub genes. (**A**) Box plot illustrating variations in immune cell infiltration between PAH and normal samples. (**B**) Heatmap of correlation between hub gene expression and immune cells. * *p* < 0.05, **** *p* < 0.0001.

CIBERSORT analysis demonstrated significant variations in immune cell profiles between PAH and normal groups by estimating the proportions of 22 immune cells in the samples (Figure 6A). PAH tissues showed a significant decrease in CD8 T cells, accompanied by a reduction in follicular T cells and activated NK cells, and an increase in neutrophils. Correlation analysis (Figure 6B) revealed that *ROCK2* and *HSP90AA1* had a strong negative correlation with CD8 T cells and activated NK cells, and a positive correlation with neutrophils. In addition, the hub genes were associated with dendritic cell–related signatures, suggesting altered activation status.

### 2.8. Correlation, Differential Expression and GSEA of Hub Genes

**Figure 7:**
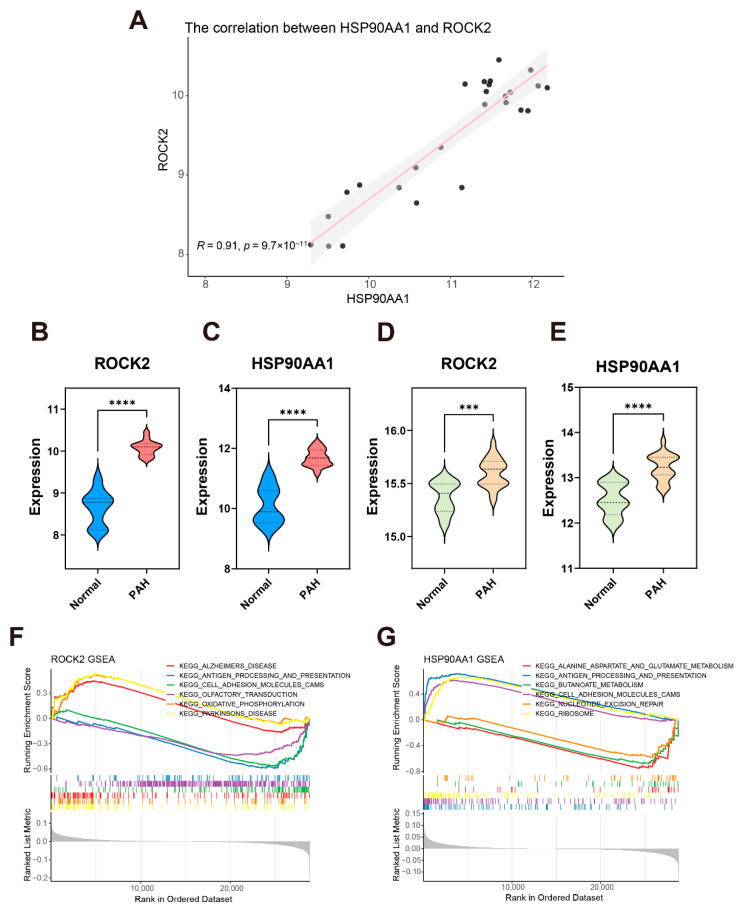
Correlation, differential expression and GSEA of hub genes. (**A**) Correlation scatter plot of *HSP90AA1* and *ROCK2*. The red line indicates the fitted regression line and the gray shaded area indicates the 95% confidence interval. (**B**,**C**) Differential expression levels of *ROCK2* and *HSP90AA1* between PAH and normal samples in the training set. (**D**,**E**) Differential expression levels of *ROCK2* and *HSP90AA1* between PAH and normal samples in the test set. (**F**,**G**) GSEA enrichment pathway plot of *ROCK2* and *HSP90AA1*. *** *p* < 0.001, **** *p* < 0.0001.

The correlation scatter plot showed a significant positive correlation between *HSP90AA1* and *ROCK2* (Figure 7A). By examining the differential expression levels of two hub genes in the training set, we found that both hub genes were notably upregulated in PAH patients (Figure 7B,C). In the test set, the expression of the two genes was also significantly upregulated in patients compared to normal samples (Figure 7D,E). The enrichment pathway plots visualized the top 3 up- and down-regulated pathways of two genes, demonstrating the functional roles of two hub genes (Figure 7F,G).

### 2.9. Single-Cell Analysis and Cellular Localization of Hub Genes

**Figure 8:**
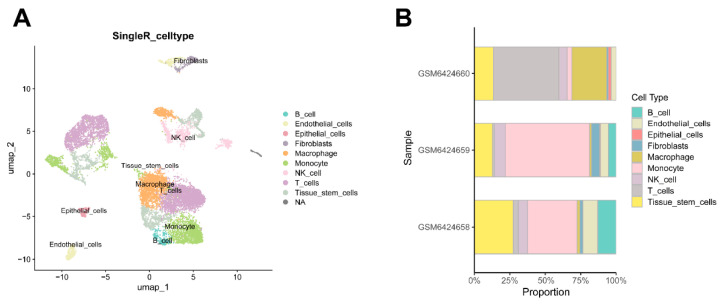
Single-cell landscape of PAH pulmonary arteries. (**A**) UMAP plot showing the annotated cell clusters. (**B**) Bar plot showing the proportional composition of cell types across three PAH samples.

To elucidate the cellular targets of CP, we examined the expression landscapes of *HSP90AA1* and *ROCK2* within the PAH microenvironment using scRNA-seq data. After rigorous quality control and dimensionality reduction, unsupervised clustering resolved the tissue into distinct lineages (Supplementary Figure S2A–D). As shown in the Uniform Manifold Approximation and Projection (UMAP) plot (Figure 8A), the cellular composition included endothelial cells, fibroblasts, tissue stem cells, and diverse immune populations, such as T cells, NK cells, macrophages, and B cells. Quantitative analysis confirmed the consistent presence of these cell types across all samples (Figure 8B).

**Figure 9:**
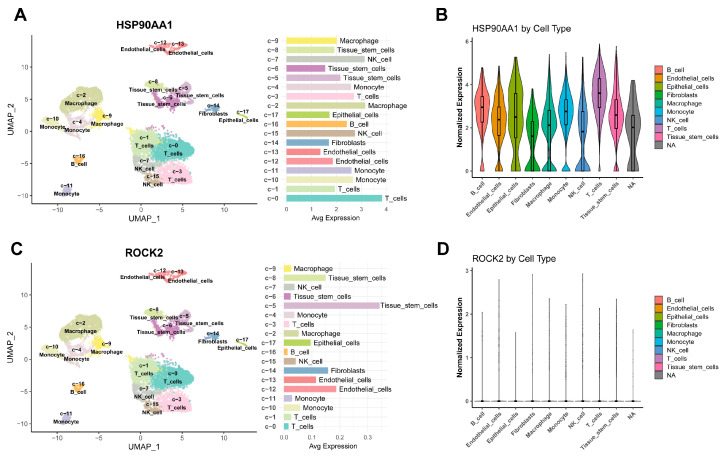
Cellular localization of hub targets *HSP90AA1* and *ROCK2*. (**A**) UMAP feature plot showing the expression level of *HSP90AA1*. (**B**) Violin plot quantifying *HSP90AA1* expression across different cell types. (**C**) UMAP feature plot for *ROCK2*. (**D**) Violin plot for *ROCK2*.

Mapping the hub genes onto these clusters revealed distinct expression patterns (Figure 9). *HSP90AA1* exhibited a broad expression profile and was enriched in immune-related clusters as well as endothelial cells, suggesting potential relevance across both immune regulation and vascular endothelial function (Figure 9A,B). In contrast, *ROCK2* showed a restricted expression pattern, predominantly localized in fibroblasts and endothelial cells rather than immune cells (Figure 9C,D). This distribution aligns with its critical role in regulating cytoskeletal reorganization, vasoconstriction, and vascular remodeling.

Given that pathological vascular remodeling is driven by the hyperproliferation of structural cells, and acknowledging the phenotypic plasticity between fibroblasts and smooth muscle cells in PAH, we hypothesized that CP might exert biological effects by influencing these vascular structural cells. Consequently, RPASMCs were selected as the in vitro model to evaluate the pharmacological effects of CP.

### 2.10. Molecular Docking of Hub Genes with Bioactive Compounds

**Figure 10:**
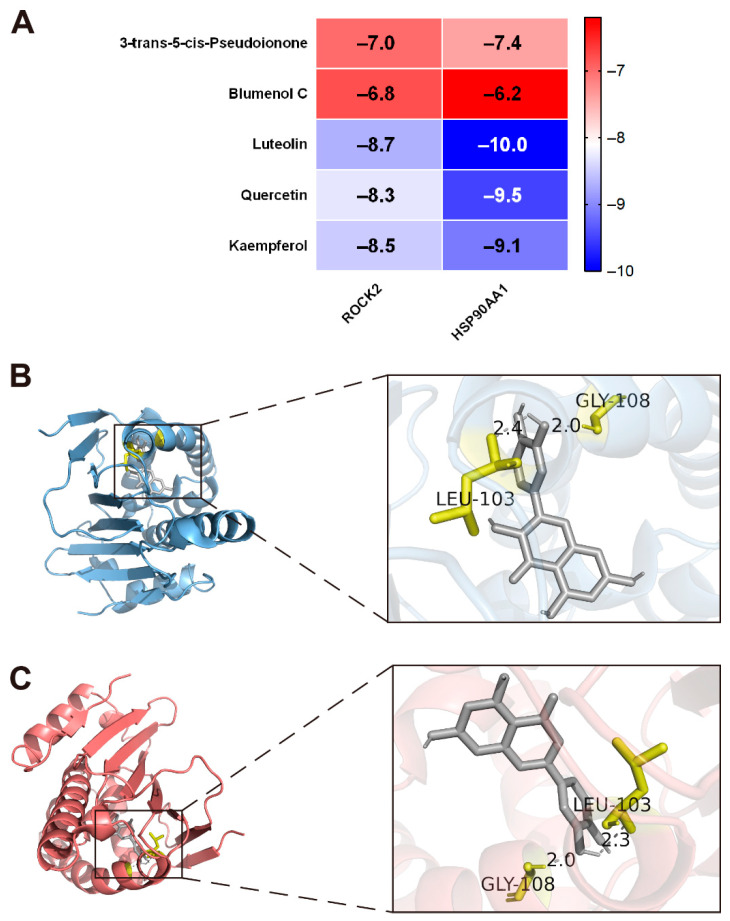
Molecular docking analysis of active components and hub genes. (**A**) Heatmap of binding energy. (**B,C**) Visualization of the docking result of quercetin–HSP90AA1 and luteolin–HSP90AA1, with the ligands shown in gray and the interacting residues shown in yellow.

Molecular docking was performed between the top 5 bioactive compounds and the hub genes to predict their binding modes and affinities. Lower docking energy indicates a more favorable predicted binding interaction. In our study, all calculated binding energies were below −6.2 kcal/mol, suggesting potential interactions between the candidate compounds and the hub targets associated with PAH (Figure 10A). Notably, luteolin, quercetin and kaempferol showed relatively higher affinities for the hub targets. Among all the results, luteolin demonstrated the strongest predicted binding affinity with HSP90AA1, with a docking energy reaching −10.0 kcal/mol. The two best docking results were visualized in Figure 10B,C.

### 2.11. Molecular Dynamics (MD) Simulation of Candidate Compounds with HSP90AA1

**Figure 11:**
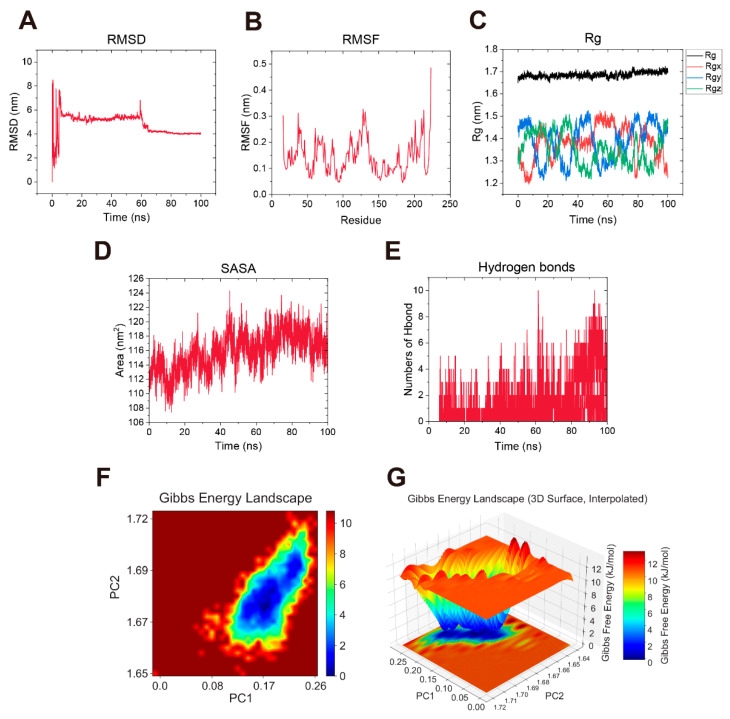
Molecular dynamics simulations of luteolin and HSP90AA1. (**A**) Root-mean-square deviation (RMSD) value of the complex. (**B**) Root-mean-square fluctuation (RMSF) values of the complex. (**C**) Radius of gyration (Rg) values of the complex. (**D**) Solvent-accessible surface area (SASA) values of the complex. (**E**) Number of hydrogen bonds in the complex. (**F**) Two-dimensional free energy landscape (2D FEL) of the complex. (**G**) Three-dimensional free energy landscape (3D FEL) of the complex.

To further evaluate binding stability, 100 ns MD simulations were performed for both HSP90AA1-quercetin and HSP90AA1-luteolin complexes. Supplementary Figure S3A showed that the RMSD of the HSP90AA1–quercetin complex exhibited significant fluctuations within 100 ns and did not reach a complete equilibrium at the end of the calculation. Furthermore, the free energy landscapes (FEL) of this complex exhibited a relatively broad energy basin across the conformational space with a free energy range of 0 to 10.5 kJ/mol, suggesting that the complex remained dynamically stable rather than rigid during the simulation (Supplementary Figure S3F,G). In contrast, the HSP90AA1–luteolin complex exhibited superior structural stability (Figure 11). Specifically, the RMSD of HSP90AA1-luteolin complex stabilized after 60 ns (Figure 11A). While both complexes maintained comparable overall compactness and local flexibility (Supplementary Figure S3B–D and Figure 11B–D), the HSP90AA1–luteolin complex formed a more robust hydrogen bond network (Figure 11E). Most notably, the HSP90AA1–luteolin complex displayed a single, tightly concentrated global energy minimum (Figure 11F,G). This concentrated basin indicates that luteolin binding significantly restricted the protein motions, effectively confining the complex to a stable dominant conformation.

### 2.12. Experimental Investigation of CP on Hypoxia-Induced RPASMCs

Our integrated computational analyses prioritized HSP90AA1 as the primary candidate target for subsequent in vitro experiments, supported by its strongest binding stability in MD simulations and extensive expression profile across relevant cell types in scRNA-seq analysis. Therefore, we focused on the in vitro evaluation of HSP90AA1-related effects in RPASMCs.

#### 2.12.1. Cytotoxicity Assessment of CP Extract

**Figure 12:**
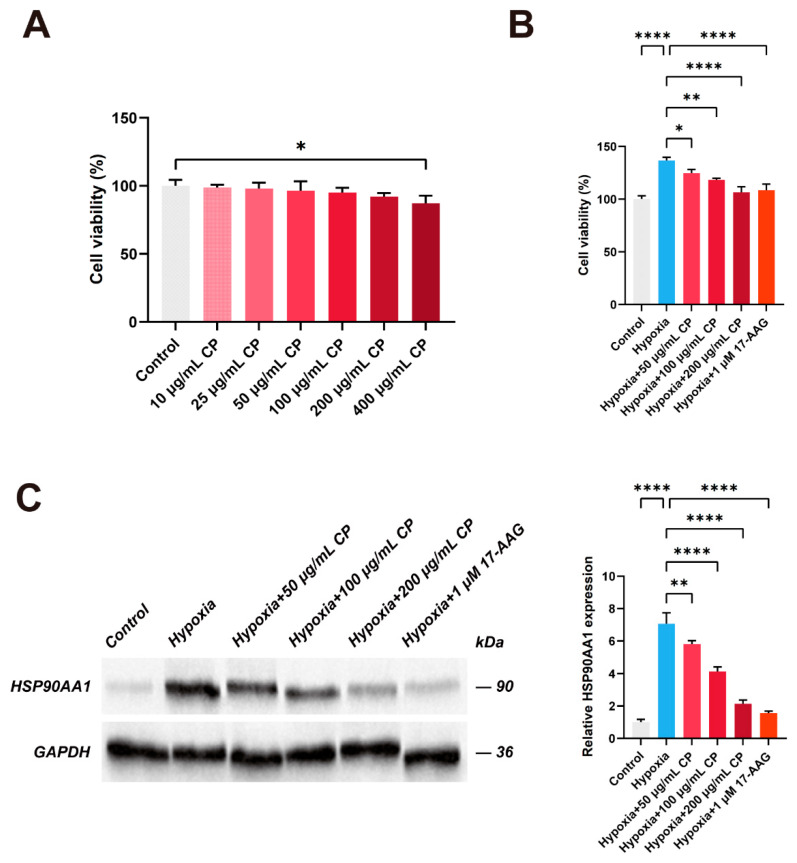
Experimental evidence of CP on RPASMCs. (**A**) Cytotoxicity assessment of CP extract on RPASMCs under normoxic conditions for 48 h. (**B**) Effect of CP extract on the viability of hypoxia-induced RPASMCs. (**C**) The protein expression levels of HSP90AA1 in RPASMCs. * *p* < 0.05, ** *p* < 0.01, **** *p* < 0.0001.

To determine the safe therapeutic concentration range, the effect of CP extract on RPASMC viability was first assessed under normoxia. Treatment with CP at concentrations ranging from 10 to 200 μg/mL for 48 h exhibited no significant cytotoxicity, whereas the highest concentration of 400 μg/mL significantly reduced cell viability (Figure 12A). A relatively high concentration range of 50–200 μg/mL was adopted for the subsequent experiment to ensure the active constituents in the crude extract could reach a sufficient threshold to induce detectable pharmacological responses.

#### 2.12.2. Effect of CP on Hypoxia-Induced RPASMCs Proliferation

Abnormal proliferation of PASMCs is a major pathological feature of PAH. To evaluate the biological effects of CP, we established a hypoxia-induced RPASMC model. As shown in Figure 12B, exposure to hypoxia for 48 h significantly increased cell viability compared to the normoxic control group. However, CP extract dose-dependently alleviated hypoxia-induced hyperproliferation, with cell viability significantly reduced at 50, 100, and 200 μg/mL compared to the untreated hypoxia group. Notably, the anti-proliferative efficacy of high-dose CP (200 μg/mL) was comparable to that of the HSP90 inhibitor, 17-allylamino-17-demethoxygeldanamycin (17-AAG, 1 μM), suggesting a potential effect of CP on PAH-related cellular changes.

#### 2.12.3. Effect of CP on HSP90AA1 Expression in Hypoxia-Induced RPASMCs

To further assess whether the antiproliferative effects of CP were associated with HSP90AA1, we examined its protein expression. Western blot analysis demonstrated a significant upregulation of HSP90AA1 in RPASMCs under hypoxic conditions (Figure 12C). Consistent with the cell viability results, intervention with CP significantly reversed this upregulation in a dose-dependent manner. The downregulation of HSP90AA1 by CP was similar to the effect observed with 17-AAG. These findings provide supportive experimental evidence that CP extract may modulate PAH-related cellular phenotypes in association with downregulation of HSP90AA1.

## 3. Discussion

Pulmonary arterial hypertension (PAH) is a fatal disease that remains a major global health challenge. Despite the development of targeted therapies for PAH, severe side effects and persistent disability remain unavoidable, accompanied by exorbitant treatment costs [4,16]. *Camellia petelotii* (Merr.) Sealy (CP) contains multiple bioactive constituents with diverse molecular targets, which makes systematic investigation necessary. Therefore, integrating computational and in vitro experimental approaches is useful for identifying candidate molecular targets and mechanisms associated with the effects of CP in PAH [17].

Our integrated analysis screened 3-trans-5-cis-pseudoionone, blumenol C, luteolin, quercetin, and kaempferol as the primary bioactive compounds of CP. Although research on the top two compounds remains limited, the cardiovascular protective effects of luteolin, quercetin, and kaempferol have been extensively substantiated, including potent anti-inflammatory, antioxidant, and vasorelaxant effects crucial for combating hypertension and vascular remodeling [18,19,20]. Through integrated differential gene expression analysis, WGCNA, and PPI analysis, we found 9 core targets and identified the “Lipid and Atherosclerosis” pathway as the most significantly enriched pathway. This suggests the potential involvement of CP in processes related to endothelial dysfunction, vascular inflammation, lipid metabolism dysregulation, and vascular remodeling, which were core mechanisms in PAH [21,22].

Machine learning algorithms pinpointed *HSP90AA1* and *ROCK2* as hub targets associated with the effects of CP in PAH. Both targets demonstrated significant upregulation in PAH patients. Immune cell infiltration analysis showed that both *HSP90AA1* and *ROCK2* were negatively correlated with CD8 T cells and activated NK cells but positively correlated with neutrophils. This pattern suggests an altered immune microenvironment in PAH, with coordinated changes in immune cell dynamics characterized by reduced cytotoxic lymphocyte signals and enhanced neutrophil-associated inflammation. Further GSEA indicated that these hub genes may be associated with cellular processes relevant to PAH pathogenesis. ROCK2, an isoform of Rho-kinase [16], is a master regulator of vascular smooth muscle cell contraction, motility, proliferation, migration, and endothelial dysfunction, promoting the progression of cardiovascular pathologies such as PAH [23,24]. It also acts as a critical metabolic and immune regulator. The enrichment of oxidative phosphorylation-related pathways suggests that ROCK2 may be involved in metabolic reprogramming in PAH [25]. HSP90AA1, a subtype of the molecular chaperone HSP90, exerts vascular remodeling effects [26,27]. HSP90 inhibitors may attenuate the development of PAH by inhibiting PDGFR activation to modulate vascular smooth muscle cell migration and proliferation [28,29]. It is also associated with multiple metabolic pathway alterations, suggesting its potential relevance to PAH metabolic reprogramming and broader pathological processes, including cell growth and stress adaptation [30,31,32]. *ROCK2* and *HSP90AA1* were respectively linked to the downregulated and upregulated antigen processing and presentation and cell adhesion molecules, suggesting their potential involvement in immune-related alterations in PAH [33,34]. Their established roles in PAH pathogenesis support the possibility that they may act as candidate molecular mediators of the effects of CP in PAH.

Single-cell transcriptomic analysis was performed to clarify the cellular localization of predicted hub targets and reveal their specific cellular context within the heterogeneous microenvironment of PAH [35]. While *HSP90AA1* is traditionally recognized as a broadly expressed molecular chaperone, our scRNA-seq analysis further characterized its distribution across immune-related and vascular structural cell populations in PAH, particularly fibroblasts and endothelial cells [36,37]. In contrast, *ROCK2* displayed a more restricted expression profile, predominantly localized within fibroblasts and endothelial cells, with no notable enrichment in any immune cell clusters. This is consistent with its critical function of regulating cytoskeletal reorganization and vasoconstriction. Importantly, the expression of both targets in vascular structural cells further supports their potential relevance to vascular remodeling in PAH. Previous studies have shown that pathological vessel narrowing is mainly driven by hyperproliferation and phenotypic switching of vascular structural cells rather than solely by immune infiltration [38,39]. Accordingly, RPASMCs were selected as the in vitro model to further examine the antiproliferative effects of CP.

To further substantiate the interactions between major constituents and the predicted targets, we performed molecular docking. The strong binding energies indicated possible binding conformations between the key CP constituents and the hub targets. Given that ubiquitous polyphenols are often classified as pan-assay interference compounds (PAINS), which may yield false-positive results in in silico models, MD simulations were performed to discriminate specific binding from transient nonspecific interactions [40,41,42]. The MD trajectories demonstrated that the complex of luteolin and HSP90AA1 achieved a rapid and sustained equilibrium in RMSD values and displayed a single, deep energy basin in the free energy landscape [43,44]. These results support a relatively stable interaction in silico and provide additional rationale for prioritizing HSP90AA1 for in vitro experiments, while ROCK2 was retained as a candidate hub target for future verification.

Subsequent in vitro experiments supported our computational predictions. Hypoxia potently induced RPASMC hyperproliferation, a primary driver of vascular remodeling in PAH [45,46]. CP extract significantly inhibited this proliferation in a dose-dependent manner, exhibiting efficacy comparable to the HSP90 inhibitor 17-AAG [47]. Additionally, hypoxia stimulation markedly upregulated HSP90AA1 protein expression, which could be reversed by CP treatment. These findings supported the anti-proliferative effect of CP in hypoxia-induced RPASMCs and were consistent with regulation of HSP90AA1-related signaling in this model [48]. However, without direct chemical characterization, the present results should be interpreted at the level of the total CP extract, and the contribution of individual constituents to these effects remains to be determined.

Despite the promising findings, certain limitations persist in this research. First, our screening of active constituents was based on published literature rather than direct phytochemical analyses. Comprehensive chemical profiling or bioactive fraction analysis, such as UPLC-Q-TOF-MS/MS, is still needed to identify the active constituents of CP more accurately. Accordingly, since the in vitro experiments used total CP extract without characterization, the flavonoids we discussed should be regarded as candidate components rather than experimentally confirmed components of the tested extract, and the tested concentrations should be interpreted as exploratory in vitro doses. Further research should isolate the predicted active monomers. Second, immune infiltration was estimated from bulk transcriptomic deconvolution and should be further confirmed by independent methods such as flow cytometry or immunostaining. Third, although in vitro evidence showed that CP inhibited PASMC proliferation and was associated with downregulation of HSP90AA1, its efficacy and safety remain to be verified in vivo. Additionally, our experimental investigation was limited to HSP90AA1. Functional validation should be extended to *ROCK2* and other candidate targets to clarify their specific contributions and the specificity of HSP90AA1-related signaling under CP treatment.

## 4. Materials and Methods

### 4.1. Acquisition and Prediction of Bioactive Compounds and Targets of CP

The compounds of CP were obtained from published literature. The “SMILES” of each compound was retrieved from the PubChem database (https://pubchem.ncbi.nlm.nih.gov/, accessed on 15 June 2025) and submitted to the SwissADME database (http://swissadme.ch, accessed on 16 June 2025) to screen bioactive constituents. Compounds were selected based on high gastrointestinal (GI) absorption and compliance with at least two drug-likeness rules [49]. The potential drug targets prediction was performed in the SwissTargetPrediction database (http://swisstargetprediction.ch, accessed on 17 June 2025). A compound-target network was constructed, and candidate compounds were prioritized according to degree values in the network.

### 4.2. DEGs Screening of PAH

PAH-related gene expression profiles from the training set GSE113439 containing fresh-frozen lung samples of 15 patients with PAH and 11 normal controls were obtained from the Gene Expression Omnibus (GEO) repository (https://www.ncbi.nlm.nih.gov/geo/, accessed on 18 June 2025). The raw transcriptome profiles were normalized and sample quality was assessed via principal components analysis (PCA) performed on the Metware Cloud platform (https://cloud.metware.cn/, accessed on 18 June 2025). Genes with threshold of |log_2_FC| > 1 and adjusted *p* value < 0.05 were identified as differential expression genes (DEGs) using the Limma R 4.5.0 package [50]. Volcano plot of differential genes and clustering heatmap were visualized through the Metware Cloud platform (accessed on 19 June 2025). The top 10 up-regulated and top 10 down-regulated genes were visualized in a histogram using Origin 2025.

### 4.3. Module Targets Screening Using WGCNA

Weighted gene co-expression network analysis was performed using the WGCNA R 4.5.0 package [51]. The adjacency matrix was transformed into a topological overlap matrix based on the optimal soft-threshold power. Then, genes were hierarchically clustered to form modules, and the correlation between modular gene expression and PAH was calculated to find the key module targets associated with PAH. The intersection of target genes of CP, DEGs and module targets was conducted and visualized by the Evenn platform (https://www.bic.ac.cn/EVenn/, accessed on 22 June 2025).

### 4.4. Construction of Protein–Protein Interaction (PPI) Network

The PPI network of the intersection targets was constructed in the STRING 12.0 database (https://string-db.org/, accessed on 25 June 2025) with a confidence interaction score threshold ≥ 0.4 and further analyzed in Cytoscape 3.10.0 to characterize the PPI network topology.

### 4.5. Enrichment Analysis

Gene Ontology (GO) biofunctional enrichment analysis and Kyoto Encyclopedia of Genes and Genomes (KEGG) pathway enrichment analysis of the overlapping genes were carried out with the clusterProfiler package [52] in R 4.5.0 software. Remarkably enriched pathways were presented as bubble charts based on the criteria of *p*-value < 0.05.

### 4.6. Hub Gene Selection Using Machine Learning Algorithms

The intersecting genes were further refined by two machine learning methods, including random forest (RF) [53] and LASSO algorithms [54], and genes within the overlap were selected as hub targets potentially associated with the effects of CP in PAH.

### 4.7. Immune Cell Infiltration Analysis

Immunity plays a critical role in the pathophysiology of PAH; thus, we conducted the immune cell infiltration analysis on the CIBERSORTx platform (https://cibersortx.stanford.edu/, accessed on 29 June 2025) [55] to examine the relationship between immune cells and DEGs. The correlation result matrix was visualized as a box plot in the Sangerbox database (http://sangerbox.com/, accessed on 30 June 2025). Correlation between hub gene expression levels and immune cell infiltration levels was calculated and visualized in R 4.5.0 software with compatible statistical packages. Statistical significance was determined at *p* < 0.05 (*) and *p* < 0.0001 (****).

### 4.8. Correlation, Differential Expression and Gene Set Enrichment Analysis (GSEA) of Hub Genes

The correlation between the two hub genes was evaluated using the correlation package in R 4.5.0 software, and a scatter plot was generated for visualization. The gene expression profiles of the test set GSE48149, which contains lung tissues of 21 patients with PAH and nine healthy people, were obtained from the GEO database. The expression levels of hub genes in patients and normal samples of both the training set and test set were visualized as violin plots in Graphpad Prism 10.1.2 and statistically analyzed. Significant differences were determined at *p* < 0.001 (***) and *p* < 0.0001 (****). Additionally, GSEA [56] was performed for each hub gene to identify their biological enrichment pathways.

### 4.9. Single-Cell RNA-Seq Analysis

To characterize the cellular landscape of PAH and identify the specific cell types targeted by CP, we analyzed a public scRNA-seq dataset GSE210248 from the GEO database (accessed on 13 November 2025). This dataset contains pulmonary artery tissues from PAH patients. The raw gene expression matrices were processed using the Seurat R package (version 4.5.2) [57]. Quality control was performed to exclude low-quality cells by filtering out cells with fewer than 200 or more than 2500 detected genes, as well as cells exhibiting mitochondrial gene content exceeding 10%. The data were normalized using the LogNormalize method, and the top 2000 highly variable genes were identified for dimensional reduction. PCA was performed, followed by Uniform Manifold Approximation and Projection (UMAP) for two-dimensional visualization. Cell clusters were annotated using the SingleR algorithm [58] with reference transcriptomic profiles from the Human Primary Cell Atlas. Finally, the expression distributions of the hub target genes, *HSP90AA1* and *ROCK2*, were visualized across distinct cell lineages using UMAP feature plots and violin plots to evaluate their cell-type specificity.

### 4.10. Molecular Docking

The 3D structures of the hub targets were retrieved from the Protein Data Bank database (https://www.rcsb.org/, accessed on 3 July 2025) as PDB files. The structures of the candidate bioactive compounds were downloaded from the PubChem database (https://pubchem.ncbi.nlm.nih.gov/, accessed on 3 July 2025) in SDF format and converted to mol2 files using OpenBabel 3.1.1. Ligand preparation was carried out in AutoDock Tools 1.5.7, including hydrogen addition, charge assignment, and rotatable bond definition, followed by conversion to pdbqt format. For receptor preparation, water molecules were removed and polar hydrogens were added before the protein files were converted into pdbqt files. Molecular docking was performed with AutoDock Vina 1.2.3 [59]. The docking results were ranked according to binding energy, and the two complexes with the lowest binding energies were selected for subsequent visualization and interaction analysis in PyMOL 3.1.3.

### 4.11. Molecular Dynamics (MD) Simulation

Based on the molecular docking results, the protein-ligand complexes exhibiting the highest binding affinity were selected for further molecular dynamics simulations in GROMACS 2020.6 [60] to assess the stability of the protein-ligand complexes. The CHARMM36 force field [61] was employed for the protein, and the TIP3P water model was utilized for system solvation. Ligand parameters were assigned via Sobtop 1.0 and transformed into GROMACS-compatible formats. MD simulations were performed as follows. First, the molecule was solvated in a predefined box filled with TIP3P water molecules to mimic the aqueous environment. Subsequently, Na+ and Cl− ions were added to neutralize the system. Energy minimization was then performed using the steepest descent algorithm to eliminate steric clashes. After that, the system was equilibrated under NVT (constant volume and temperature) at 310K and NPT (constant pressure and temperature) ensembles at 1 bar. Finally, the simulation spanning 100 ns was conducted. The trajectory files were processed to remove periodic boundary artifacts, followed by calculation of the protein-ligand root-mean-square deviation (RMSD), protein root-mean-square fluctuation (RMSF), radius of gyration (Rg), solvent accessible surface area (SASA), and number of hydrogen bonds (H-bonds). Origin 2025 was then utilized to visualize the results. Additionally, the free energy landscape (FEL) was constructed to assess the thermodynamic stability of the complexes. The covariance matrix of the backbone Cα atoms was analyzed to obtain the principal components (PCs). The first two PCs (PC1 and PC2) were selected as reaction coordinates to map the conformational space. The Gibbs free energy (Δ*G*) was calculated using the formula $\Delta G\left(V\right)=-k_{B}T\ln P(V)$, where *p*(*V*) represents the probability distribution of the system along the reaction coordinates.

### 4.12. In Vitro Experiments

#### 4.12.1. Materials and Reagents

The flowers of *Camellia petelotii* (Merr.) Sealy (CP20250312) were provided by the Guangxi Institute of Botany, Chinese Academy of Sciences (Guilin, China), and were collected in Guangxi Province, China, in March 2025. The plant material was authenticated by Prof. Zhirong Sun (Beijing University of Chinese Medicine, Beijing, China) and the sample (CP20250312) was deposited in the Laboratory of Traditional Chinese Medicine Resources, Beijing University of Chinese Medicine. Dried flower material (10 g) was extracted twice with 80% ethanol (1:10, *w*/*v*) under reflux for 2 h each time. The combined filtrates were concentrated and freeze-dried to obtain the crude extract, with an extraction yield of 28.47% (*w*/*w*).

Fetal bovine serum (FBS; Cat. No. SH30070.03) was purchased from HyClone (Logan, UT, USA), penicillin-streptomycin (Cat. No. 15140148) was purchased from Thermo Fisher Scientific (Pittsburgh, PA, USA), and Dulbecco’s Modified Eagle Medium (DMEM; Cat. No. 10566016) were obtained from Gibco (Grand Island, NY, USA). The Cell Counting Kit-8 (CCK-8) was obtained from Beyotime Biotechnology (Shanghai, China). The HSP90 inhibitor 17-allylamino-17-demethoxygeldanamycin (17-AAG; Cat. No. S1141) was purchased from Selleckchem (Houston, TX, USA) and used as a positive control. Primary antibodies against HSP90AA1 (Cat. No. 4874) were purchased from Cell Signaling Technology (Boston, MA, USA), and GAPDH (Cat. No. ab8245) was obtained from Abcam (Cambridge, UK). The HRP-conjugated goat anti-rabbit IgG secondary antibody (Cat. No. ab205718) was purchased from Abcam. RIPA lysis buffer (Cat. No. KGP702-100) and the bicinchoninic acid (BCA) assay kit (Cat. No. KGP902) were obtained from KeyGEN BioTECH (Nanjing, China). Phenylmethylsulfonyl fluoride (PMSF; Cat. No. 97064-898) was purchased from Amresco, VWR International (Solon, OH, USA). Ten percent SDS (Cat. No. BL517A) was obtained from Biosharp (Hefei, China). Polyvinylidene fluoride (PVDF) membranes (0.2 μm; Cat. No. ISEQ00010) were purchased from Millipore (Schwalbach, Germany), and TBST powder (Cat. No. G0001) was obtained from Servicebio (Wuhan, China). The enhanced chemiluminescence (ECL) reagent kit (Cat. No. 32209) was purchased from Thermo Fisher Scientific (Pittsburgh, PA, USA).

#### 4.12.2. Cell Culture and Hypoxia Model Establishment

RPASMCs were purchased from WHELAB (Shanghai, China) and cultured in DMEM supplemented with 10% FBS, 100 U/mL penicillin, and 100 μg/mL streptomycin at 37 °C in a humidified atmosphere containing 5% CO_2_.

For the hypoxia model, cells were incubated in a hypoxic chamber containing 1% O_2_, 5% CO_2_, and 94% N_2_ [62]. The control group was maintained under normoxic conditions (95% air, 5% CO_2_).

#### 4.12.3. Cell Viability Assay

Cell viability was assessed using the CCK-8 assay. RPASMCs were seeded into 96-well plates at a density of 1 × 10^4^ cells/well. To determine the safe dose range under normoxic conditions, cells were treated with increasing concentrations of CP extract (0, 10, 25, 50, 100, 200, and 400 μg/mL) for 48 h. To evaluate efficacy, cells were exposed to hypoxia and treated with selected non-cytotoxic concentrations of CP extract (50, 100, and 200 μg/mL) or the positive control 17-AAG (1 μM) for 48 h. After treatment, 100 μL of fresh medium containing 10% (*v*/*v*) CCK-8 solution was added to each well and incubated for 2 h at 37 °C. Absorbance was measured at 450 nm using a microplate reader.

#### 4.12.4. Western Blot

Total protein was extracted from RPASMCs using RIPA lysis buffer supplemented with PMSF, and protein concentration was determined using the BCA assay. Protein samples (60 μg) were separated by 10% SDS-PAGE and transferred onto PVDF membranes. The membranes were blocked with 5% non-fat milk and incubated overnight at 4 °C with primary antibodies against HSP90AA1 (1:1000) and GAPDH (1:10,000). After washing with TBST, the membranes were incubated with HRP-conjugated secondary antibodies (1:10,000) for 1 h at room temperature. Protein bands were visualized using an ECL reagent kit and quantified using Image-Pro Plus 6.0 software.

#### 4.12.5. Statistical Analysis

All experimental data are presented as mean ± SD from at least three independent experiments. Statistical differences were analyzed using one-way or two-way analysis of variance (ANOVA) with GraphPad Prism 10.1.2 software. Values of *p* < 0.05 (*), *p* < 0.01 (**), and *p* < 0.0001 (****) were used to determine significant differences.

## 5. Conclusions

In conclusion, this study explored the potential molecular basis underlying the effects of *Camellia petelotii* (Merr.) Sealy in pulmonary arterial hypertension by integrating computational prediction with experimental evidence. Using network pharmacology, WGCNA, and machine learning algorithms, we prioritized luteolin and quercetin as candidate bioactive constituents of CP and identified *HSP90AA1* and *ROCK2* as hub targets. Furthermore, single-cell transcriptomic analysis of *HSP90AA1* and *ROCK2* in PAH provided additional insight into their disease-relevant cellular localization and potential roles in PAH. Subsequently, molecular docking and molecular dynamics simulations suggested a relatively stable interaction between luteolin and HSP90AA1 in silico, while in vitro experiments in RPASMCs showed that CP extract inhibited hypoxia-induced cell proliferation and downregulated HSP90AA1 protein expression. Together, these results support HSP90AA1 as a candidate molecular mediator of CP extract in PAH and warrant further investigation of CP and its predicted active constituents.

## Figures and Tables

**Table 1:** Candidate bioactive compounds of CP.

Code	Compound Name	Code	Compound Name	Code	Compound Name
JHC1	Phloretin	JHC19	Guaiol	JHC37	trans-5-Methyl-2-isopropyl-2-hexen-1-al
JHC2	2-Pentylfuran	JHC20	Agaruspirol	JHC38	Benzeneacetaldehyde
JHC3	Theobromine	JHC21	Lupeol	JHC39	Methyl salicylate
JHC4	Vanillin	JHC22	Oleanolic acid	JHC40	2-Isopropenyl-5-methylhex-4-enal
JHC5	Caffeine	JHC23	Phytolaccagenin	JHC41	cis-3-Hexenyl benzoate
JHC6	Geranyl acetone	JHC24	Asiatic acid	JHC42	Phenylethyl alcohol
JHC7	Nerol	JHC25	Ginsenoside F1	JHC43	(R)-(−)-1-Phenyl-1,2-ethanediol
JHC8	trans-Geraniol	JHC26	Arjunolic acid	JHC44	Daucic acid
JHC9	Safranal	JHC27	Maslinic acid	JHC45	Shikimic acid
JHC10	Eucalyptol	JHC28	Glycyrrhetinic acid	JHC46	Dodecanoic acid
JHC11	trans-Farnesol	JHC29	Stigmasterol	JHC47	(R,S)-5-Ethyl-6-methyl-3E-hepten-2-one
JHC12	Zerumbone	JHC30	Daucosterol	JHC48	3-trans-5-cis-Pseudoionone
JHC13	Elemol	JHC31	(+)-Catechin	JHC49	Quercetin
JHC14	a-Ionone	JHC32	Coumarin	JHC50	Kaempferol
JHC15	Blumenol C	JHC33	Eudesmin	JHC51	Luteolin
JHC16	Juniper camphor	JHC34	5-Methylfurfural	JHC52	3-Methyl-6-hydroxy-8-methoxy-3,4-dihydroisocoumarin
JHC17	Dehydrololiolide	JHC35	Benzaldehyde	JHC53	Gallocatechin gallate
JHC18	Nootkatone	JHC36	2-Formylfuran	JHC54	Rutin

## Data Availability

The data presented in this study are openly available in Gene Expression Omnibus at https://www.ncbi.nlm.nih.gov/geo/ (accessed on 18 June 2025), reference number GSE113439, GSE48149, and GSE210248.

## Supplementary Materials

The following supporting information can be downloaded at: https://www.mdpi.com/article/10.3390/ijms27083687/s1. References [8,9,10,63,64,65,66,67,68,69,70,71,72,73,74,75,76,77,78,79,80,81,82,83,84,85,86] are cited in the supplementary materials.

## Abbreviations

The following abbreviations are used in this manuscript:

CP	*Camellia petelotii* (Merr.) Sealy
PAH	pulmonary arterial hypertension
WGCNA	Weighted gene co-expression network analysis
LASSO	Least absolute shrinkage and selection operator
GO	Gene Ontology
KEGG	Kyoto Encyclopedia of Genes and Genomes
GSEA	Gene set enrichment analysis
scRNA-seq	Single-cell RNA sequencing
MD	Molecular dynamics
RPASMCs	Rat pulmonary artery smooth muscle cells
GEO	Gene Expression Omnibus
DEGs	Differential expression genes
PPI	Protein–protein interaction
TCM	Traditional Chinese Medicine
PCA	Principal components analysis
BP	Biological processes
CC	Cellular component
MF	Molecular function
UMAP	Uniform Manifold Approximation and Projection
RMSD	Root-mean-square deviation
RMSF	Root-mean-square fluctuation
Rg	Radius of gyration
SASA	Solvent accessible surface area
FEL	Free energy landscape
CCK-8	Cell counting kit-8
17-AAG	17-allylamino-17-demethoxygeldanamycin
PVDF	Polyvinylidene fluoride
JHC	Jinhuacha
PAINS	Pan-assay interference compounds
