# Supplementary material for: Integrated Bioinformatics Analysis and In Vitro Evidence Support HSP90AA1 as a Candidate Target of Camellia petelotii (Merr.) Sealy in Pulmonary Arterial Hypertension
Source: Int J Mol Sci. 2026 Apr 21;27(8):3687. doi: 10.3390/ijms27083687 (PMC13116764; doi:10.3390/ijms27083687)
Supplement: Supplementary file 1 [file ijms-27-03687-s001.zip › Supplementary Figure.pdf]

Supplementary Figure

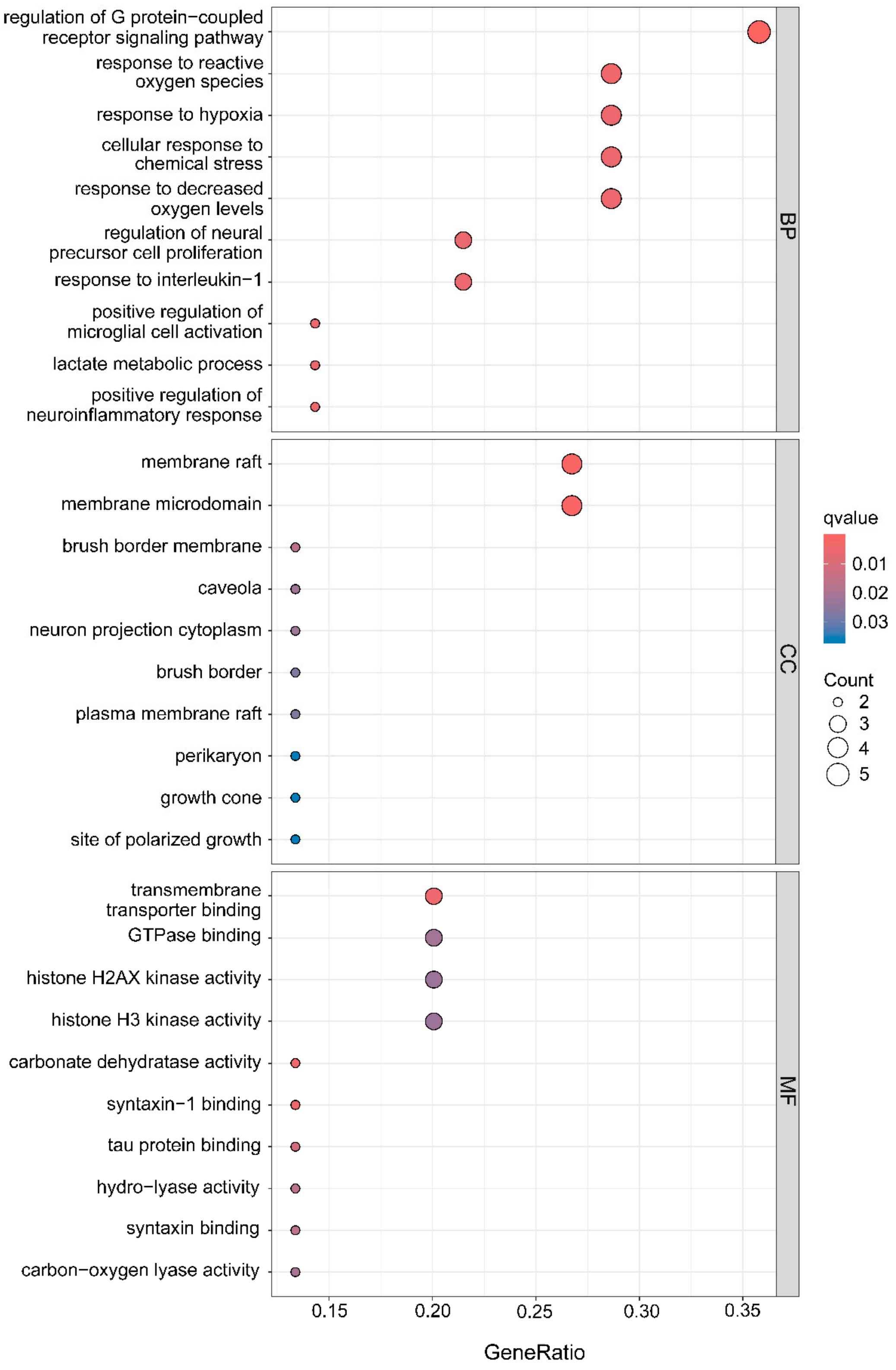

Figure S1. GO enrichment analysis

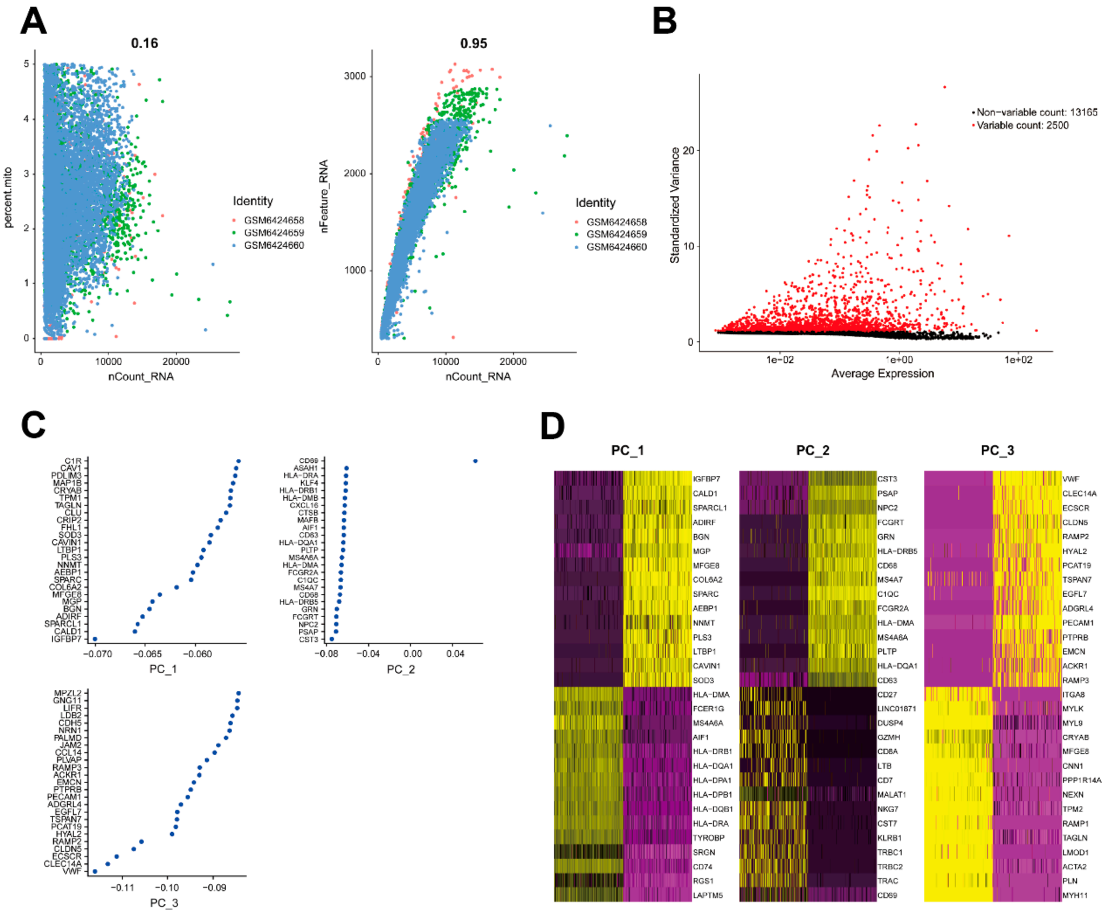

Figure S2. Single-cell landscape of PAH pulmonary arteries. (A) Quality control metrics showing nFeature, nCount, and mitochondrial percentage. (B) Variance analysis identifying top variable genes. (C) PCA plot of gene loadings. (D) Heatmap of top PC gene signatures.

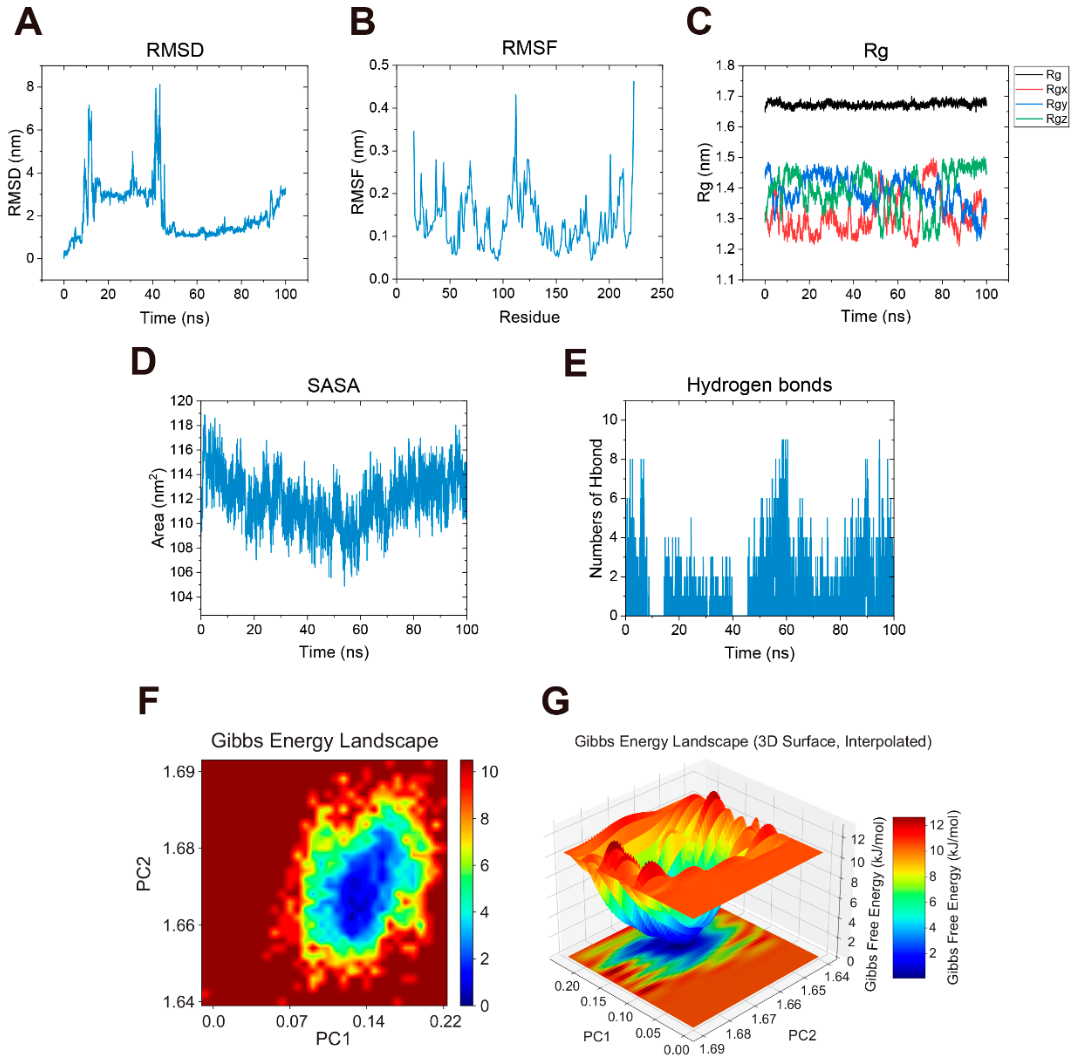

Figure S3. Molecular dynamics simulations of quercetin and HSP90AA1. (A) RMSD values of the complex. (B) RMSF values of the complex. (C) Rg values of the complex. (D) SASA values of the complex. (E) Number of hydrogen bonds in the complex. (F) Two-dimensional (2D) FEL of the complex. (G) Three-dimensional (3D) FEL of the complex.
