# Supplementary material for: Integrated Bioinformatics Analysis and In Vitro Evidence Support HSP90AA1 as a Candidate Target of Camellia petelotii (Merr.) Sealy in Pulmonary Arterial Hypertension
Source: Int J Mol Sci. 2026 Apr 21;27(8):3687. doi: 10.3390/ijms27083687 (PMC13116764; doi:10.3390/ijms27083687)
Supplement: Supplementary file 1 [file ijms-27-03687-s001.zip › Supplementary Table S1.pdf]

Supplementary Table S1 Chemical composition Schedule

No	Name	Molecular	MW	Tissues	References
1	Quercetin	$C_{15}H_{10}O_7$	302.23	Flower	[1]
2	Kaempferol	$C_{15}H_{10}O_6$	286.24	Flower	[1]
3	Pollenitin	$C_{16}H_{12}O_7$	316.26	Flower	[1]
4	Luteolin	$C_{15}H_{10}O_6$	286.24	Flower	[2]
5	(−)-Catechin	$C_{15}H_{14}O_6$	290.27	Leaf, Flower	[1,3]
6	(+)-catechin	$C_{15}H_{14}O_6$	290.27	Flower	[4]
7	(−)-Epicatechin	$C_{15}H_{14}O_6$	290.27	Whole	[2]
8	Epigallocatechin	$C_{15}H_{14}O_7$	306.27	Flower	[5]
9	Aromadendrin	$C_{15}H_{12}O_6$	288.25	Leaf	[2]
10	Taxifolin	$C_{15}H_{12}O_7$	304.25	Leaf	[6]
11	Gallocatechin gallate	$C_{22}H_{18}O_{11}$	458.37	Leaf	[7]
12	(−)-Epicatechin gallate	$C_{22}H_{18}O_{10}$	442.37	Leaf	[7]
13	Epigallocatechin gallate	$C_{22}H_{18}O_{11}$	458.37	Leaf	[7]
14	(−)-Catechin gallate	$C_{22}H_{18}O_{10}$	442.37	Leaf	[7]
15	Phloretin	$C_{15}H_{14}O_5$	274.27	Leaf	[6]
16	Procyanidin B3	$C_{30}H_{26}O_{12}$	578.52	Whole	[2]
17	Procyanidin C1	$C_{45}H_{38}O_{18}$	866.77	Whole	[2]
18	Quercetin-3-O-β-D-glucopyranoside	$C_{21}H_{20}O_{12}$	464.40	Flower	[8]
19	Kaempferol-3-O-[2-O-(*trans*-p-coumaroyl)-3-α-D-Glucopyranosyl]-α-D-glucopyranoside	$C_{36}H_{36}O_{18}$	756.67	Leaf	[9]
20	Rutin	$C_{27}H_{30}O_{16}$	610.52	Flower	[8]
21	Kaempferol-3-O-β-D-glucopyranosyl	$C_{21}H_{20}O_{11}$	448.38	Flower	[1]

22	Kaempferol-3-O-β-D-glucopyranoside	$C_{21}H_{20}O_{11}$	448.38	Flower	[8]
23	Kaempferol-3-O-β-D-rutinoside	$C_{27}H_{30}O_{15}$	594.52	Flower	[1]
24	Hyperoside	$C_{21}H_{20}O_{12}$	464.38	Leaf	[10]
25	Multiflorin C	$C_{29}H_{32}O_{16}$	636.56	Flower	[1]
26	Kaempferol-3-O-(6′′-O-*trans*-p-cou-maroyl)-β-D- glucopyranoside	$C_{30}H_{26}O_{13}$	594.53	Flower	[11]
27	Kaempferol-3-O-[α-L-Rhamnopyranosyl-(1→3)-2,4-di-O-acetyl-α-L-rham-nopyranosyl-(1→6)]-β-D-Glucopyranoside	$C_{37}H_{44}O_{21}$	825.81	Flower	[1]
28	Kaempferol-3-O-[4-O-Acetyl-α-L-rhamnopyranosyl-(1→3)-2,4-di-O-ace-tyl-α-L-rhamnopyranosyl-(1→6)]-β-D-glucopyranoside	$C_{39}H_{46}O_{22}$	890.77	Flower	[1]
29	Kaempferol-3-O-[2,3,4-Tri-O-acetyl-α-L-rhamnopyranosyl-(1→3)-4-O-acetyl-α-L-rhamnopyranosyl-(1→6)]-β-D-glucopyranoside	$C_{41}H_{48}O_{23}$	932.80	Flower	[1]
30	Kaempferol-3-O-[2,3,4-Tri-O-acetyl-α-L-rhamnopyranosyl-(1→3)-2,4-di-O-acetyl-α-L-rhamnopyranosyl-(1→6)]-β-D-glucopyranoside	$C_{43}H_{50}O_{24}$	974.83	Flower	[1]
31	Quercetin-glucosyl-rhamnosyl-glucoside	$C_{33}H_{40}O_{21}$	772.66	Flower	[1]
32	Kaempferol-glucosyl-rhamnosyl-glucoside	$C_{33}H_{40}O_{20}$	756.66	Flower	[1]
33	Kaempferol-3-O-rhamnoside	$C_{21}H_{20}O_{10}$	432.38	Flower	[11]
34	Sexangularetin 3-O-(2′′-*trans*-p-cou-marolyglucopyranoside)	$C_{31}H_{28}O_{14}$	624.55	Flower	[11]
35	3-O-Neohesperidoside-7-Rha Kaempferol	$C_{33}H_{40}O_{19}$	740.66	Whole	[2]
36	Kaempferol-3-O-β-D-glucopyranoside	$C_{42}H_{46}O_{23}$	918.81	Flower	[8]
37	Sexangularetin 3-O-(6′′-*trans*-p-cou-marolyglucopyranoside)	$C_{31}H_{28}O_{14}$	624.55	Flower	[11]
38	Apiin	$C_{26}H_{28}O_{14}$	564.49	Flower	[12]
39	Quercetin-7-O-β-D- glucopyranoside	$C_{21}H_{20}O_{12}$	464.38	Flower	[8]
40	Quercetin-7-O-(6"-O-E-caffeoyl)-β-D-glucopyranoside	$C_{30}H_{26}O_{15}$	626.52	Flower	[13]
41	Vitexin-7-O-β-D-glucopyranoside	$C_{27}H_{30}O_{16}$	590.52	Leaf	[14]
42	Luteolin-7-O-β-D-glucopyranoside	$C_{21}H_{20}O_{11}$	448.38	Leaf	[14]

43	kaempferol-7-O-β-D-glucoside	$C_{21}H_{20}O_{11}$	448.38	Flower	[11]
44	Naringoside	$C_{27}H_{32}O_{14}$	580.54	Whole	[2]
45	Genistein 7-glucoside	$C_{21}H_{20}O_{10}$	432.38	Whole	[2]
46	Vicenin-3	$C_{26}H_{28}O_{14}$	564.49	Whole	[2]
47	Isoschaftoside	$C_{26}H_{28}O_{14}$	564.49	Flower	[10]
48	Corymboside	$C_{26}H_{28}O_{14}$	564.50	Whole	[2]
49	Nitidissimol B	$C_{42}H_{38}O_{21}$	878.74	Flower	[11]
50	Nitidissimol D	$C_{23}H_{32}O_{10}$	468.50	Flower	[11]
51	Apigenin 7-O-rutinoside	$C_{27}H_{30}O_{14}$	578.52	Whole	[2]
52	Astilbin	$C_{21}H_{22}O_{11}$	450.39	Whole	[2]
53	Nitidissimol A	$C_{60}H_{50}O_{23}$	1139.04	Flower	[11]
54	Nitidissimol C	$C_{15}H_{20}O_{10}$	360.32	Flower	[11]
55	Luteolin 7-rutinoside-4′-glucoside	$C_{33}H_{40}O_{20}$	756.66	Whole	[2]
56	Phlorizin 4′-O-β-D-glucopyranoside	$C_{21}H_{24}O_{10}$	436.41	Leaf	[9]
57	Vitexin	$C_{21}H_{20}O_{10}$	432.38	Flower	[8]
58	Kaempferol 3-sophoroside-7-rhamnoside	$C_{33}H_{40}O_{20}$	756.66	Whole	[2]
59	Vicenin-2	$C_{27}H_{30}O_{15}$	594.52	Whole	[2]
60	Saponarin	$C_{27}H_{30}O_{15}$	594.52	Whole	[2]
61	Sarmenoside III	$C_{42}H_{46}O_{23}$	918.81	Whole	[2]
62	Luteolin-7-O-β-D-glucopyranoside	$C_{36}H_{36}O_{18}$	756.67	Whole	[2]
63	Multiflorin B	$C_{27}H_{30}O_{15}$	594.52	Leaf	[15]
64	Camelliaside A	$C_{33}H_{40}O_{20}$	756.66	Whole	[2]
65	*trans*-5-Methyl-2-isopropyl-2-hexen-1-al	$C_{10}H_{18}O$	154.25	Leaf	[16]
66	(R,S)-5-Ethyl-6-methyl-3E-hepten-2-one	$C_{10}H_{18}O$	154.25	Leaf	[16]
67	2-Isopropenyl-5-methylhex-4-enal	$C_{10}H_{16}O$	152.24	Flower	[16]

68	Linalool	$C_{10}H_{18}O$	154.25	Leaf, Flower	[16]
69	3-*trans*-5-*cis*-Pseudoionone	$C_{13}H_{20}O$	192.30	Leaf	[16]
70	Geranyl acetone	$C_{13}H_{22}O$	194.32	Leaf	[16]
71	Nerol	$C_{10}H_{18}O$	154.25	Leaf, Flower	[16]
72	*trans*-Geraniol	$C_{10}H_{18}O$	154.25	Leaf, Flower	[16]
73	*cis*-Linaloloxide	$C_{10}H_{18}O_2$	170.25	Leaf	[17]
74	β-Cyclocitral	$C_{10}H_{16}O$	152.24	Leaf	[16]
75	Safranal	$C_{10}H_{14}O$	150.22	Leaf	[16]
76	α-Cyclocitral	$C_{10}H_{16}O$	152.24	Leaf	[16]
77	Eucalyptol	$C_{10}H_{18}O$	154.25	Leaf	[6]
78	α-Terpinol	$C_{10}H_{18}O$	154.25	Leaf, Flower	[16]
79	Catalposide	$C_{22}H_{26}O_{12}$	482.44	Flower	[18]
80	Loganin	$C_{17}H_{26}O_{10}$	390.39	Leaf	[6]
81	*trans*-Nerolidol	$C_{15}H_{26}O$	222.37	Leaf, Flower	[16]
82	*trans*-Farnesol	$C_{15}H_{26}O$	222.37	Leaf	[16]
83	Zerumbone	$C_{15}H_{22}O$	218.34	Leaf	[6]
84	Elemol	$C_{15}H_{26}O$	222.37	Flower	[16]
85	Epoxy-β-ionone	$C_{13}H_{18}O_2$	206.29	Leaf	[16]
86	*cis*-β-Damascenone	$C_{13}H_{18}O$	190.29	Leaf	[16]
87	a-Ionone	$C_{13}H_{20}O$	192.30	Leaf	[16]
88	(3R,6R,7E) -3-hydroxy-4,7-megastigmadien-9-one	$C_{13}H_{20}O_2$	208.30	Leaf	[9]
89	*trans*-β-Ionone	$C_{13}H_{20}O$	192.30	Leaf	[16]
90	Blumenol C	$C_{13}H_{22}O_2$	210.32	Flower	[18]
91	β-Damascenone	$C_{13}H_{18}O$	190.29	Leaf	[19]
92	Dehydro-β-ionone	$C_{13}H_{18}O$	190.29	Leaf	[16]

93	5-epi-7-epi-α-Eudesmol	$C_{15}H_{26}O$	222.37	Flower	[16]
94	Neo-Intermedeol	$C_{15}H_{26}O$	222.37	Flower	[16]
95	α-Eudesmol	$C_{15}H_{26}O$	222.37	Flower	[16]
96	Juniper camphor	$C_{15}H_{26}O$	222.37	Flower	[16]
97	Dehydrololiolide	$C_{11}H_{14}O_3$	194.23	Flower	[18]
98	Nootkatone	$C_{15}H_{22}O$	218.34	Leaf	[6]
99	10-epi-γ-Eudesmol	$C_{15}H_{26}O$	222.37	Flower	[16]
100	γ-Eudesmol	$C_{15}H_{26}O$	222.37	Flower	[16]
101	*cis*-β-Guaiene	$C_{15}H_{24}$	204.36	Flower	[16]
102	Guaiol	$C_{15}H_{28}O$	224.39	Flower	[16]
103	Agaruspirol	$C_{15}H_{26}O$	222.37	Flower	[16]
104	α-Guaiene	$C_{15}H_{24}$	204.36	Flower	[16]
105	Lupeol	$C_{30}H_{50}O$	426.73	Flower	[8]
106	Oleanolic acid	$C_{30}H_{48}O_3$	456.71	Flower	[8]
107	oleanolic acid 3-acetate	$C_{32}H_{50}O_4$	498.75	Leaf	[20]
108	17,3β-acetoxy-20-lupanol	$C_{31}H_{52}O_3$	472.75	Leaf	[20]
109	PhytolaCPiagenin	$C_{31}H_{48}O_7$	532.72	Whole	[2]
110	Asiatic acid	$C_{30}H_{48}O_5$	488.71	Whole	[2]
111	3β,6α,13β-trihydroxyolean-7-one	$C_{30}H_{50}O_4$	474.73	Leaf	[3]
112	3β-acetoxy-20-lupanol	$C_{32}H_{54}O_3$	486.78	Leaf	[3]
113	A1-barrigenol-22a-angelate	$C_{35}H_{56}O_6$	572.83	Leaf	[3]
114	Ginsenoside Rg1	$C_{42}H_{72}O_{14}$	801.02	Leaf	[20]
115	Ginsenoside Rd	$C_{48}H_{82}O_{18}$	947.17	Leaf	[20]
116	Ginsenoside F5	$C_{41}H_{70}O_{13}$	770.99	Leaf	[20]
117	Ginsenoside F1	$C_{36}H_{62}O_9$	638.88	Leaf	[20]

118	(3β,6α,12β)-3,6,12-trihydroxydammar-24-en-20-yl-2-O-β-D-Glucopyranosyl-(2→1)-O-β-D-glucopyranosyl-(2→1)-O-α-L-rhamnopyranoside	$C_{48}H_{82}O_{18}$	947.17	Leaf	[20]
119	Camelliaolean B	$C_{30}H_{52}O_5$	492.74	Whole	[2]
120	Campetelosides A	$C_{41}H_{63}O_{15}$	795.94	Leaf	[21]
121	Campetelosides B	$C_{43}H_{65}O_{16}$	837.98	Leaf	[21]
122	Campetelosides C	$C_{43}H_{66}O_{14}$	806.99	Leaf	[21]
123	Campetelosides D	$C_{47}H_{70}O_{17}$	907.06	Leaf	[21]
124	Campetelosides E	$C_{42}H_{65}O_{17}NaS$	897.01	Leaf	[21]
125	Chikusetsusaponin IVa	$C_{42}H_{66}O_{14}$	794.98	Leaf	[21]
126	Umbellatoside B	$C_{48}H_{76}O_{19}$	957.12	Leaf	[21]
127	Silvioside E	$C_{48}H_{78}O_{18}$	943.13	Leaf	[21]
128	21β,22α-di-O-angeloyl-15α,16α,28-trihydroxyolean-12-ene 3β-O-α-L-rhamnopyranosyl-(1→3)-α-D-xylopyranosyl-(1→3)-β-D-glucopyranoside	$C_{57}H_{88}O_{22}$	1125.31	Leaf	[22]
129	Soyasaponin I	$C_{48}H_{78}O_{18}$	943.13	Leaf	[6]
130	Arjunolic acid	$C_{30}H_{48}O_5$	488.71	Leaf	[6]
131	Maslinic acid	$C_{30}H_{48}O_4$	472.71	Leaf	[6]
132	β-Amyrin	$C_{30}H_{50}O$	426.73	Leaf	[23]
133	Glycyrrhetinic acid	$C_{30}H_{46}O_4$	470.69	Flower	[18]
134	Betulin	$C_{30}H_{50}O_2$	442.73	Leaf	[6]
135	Ilexside II	$C_{47}H_{76}O_{18}$	929.11	Leaf	[14]
136	β-sitosterol	$C_{29}C_{50}O$	414.72	Flower	[8]
137	Stigmasterol	$C_{29}C_{48}O$	412.69	Flower	[18]
138	Daucosterol	$C_{35}C_{60}O_6$	576.86	Flower	[8]

139	Stigmasta-7,22-diene-3-O-[α-L-arabinopyranosyl(1→2)]-β-D-galactopyranoside	$C_{41}C_{68}O_{10}$	720.99	Leaf	[9]
140	α-spinasteryl-β-D-glucopyranoside	$C_{35}C_{58}O_6$	574.84	Leaf	[9]
141	p-Coumaric acid	$C_9H_8O_3$	164.16	Leaf	[6]
142	3-Methoxycinnamic acid	$C_{10}H_{10}O_3$	178.19	Leaf	[6]
143	Ferulic acid	$C_{10}H_{10}O_4$	194.19	Flower	[18]
144	3-Methyl-6-hydroxy-8-methoxy-3,4-dihydroisocoumarin	$C_{11}H_{12}O_4$	208.21	Leaf	[6]
145	Coumarin	$C_9H_6O_2$	146.14	Leaf	[6]
146	Threo-guaia-cylglycerol-O-4′-coniferyl ether	$C_{20}H_{24}O_7$	376.41	Flower	[11]
147	Erythro-guaiacylglycerol-O-4′-coniferyl ether	$C_{20}H_{24}O_7$	376.41	Flower	[11]
148	Eudesmin	$C_{22}H_{26}O_6$	386.44	Flower	[18]
149	Okicamelliaside	$C_{21}H_{16}O_{13}$	476.35	Whole	[2]
150	6-O-Acetylacteoside	$C_{31}H_{38}O_{16}$	666.63	Flower	[12]
151	Fraxin	$C_{16}H_{18}O_{10}$	370.31	Leaf	[6]
152	Citrusin B	$C_{27}H_{36}O_{13}$	568.57	Flower	[12]
153	Platyphylloside	$C_{25}H_{32}O_{10}$	492.52	Leaf	[24]
154	5-methylfurfural	$C_6H_6O_2$	110.11	Flower	[25]
155	3-Formylfuran	$C_5H_4O_2$	96.09	Leaf	[19]
156	Benzaldehyde	C_7H_6O	106.12	Leaf, Flower	[16]
157	2-Formylfuran	$C_5H_4O_2$	96.09	Flower	[16]
158	2-Pentylfuran	$C_9H_{14}O$	138.21	Leaf	[16]
159	*trans*-2-(2-Pentenyl)furan	$C_9H_{12}O$	136.19	Leaf	[16]
160	Benzeneacetaldehyde	C_8H_8O	120.15	Flower	[16]
161	Methyl salicylate	$C_8H_8O_3$	152.15	Leaf, Flower	[16]
162	Vanillin	$C_8H_8O_3$	152.15	Flower	[8]

163	p-Isopropylbenzyl alcohol	$C_{10}H_{14}O$	150.22	Flower	[16]
164	*cis*-3-Hexenyl benzoate	$C_{13}H_{16}O_2$	204.27	Leaf	[16]
165	Benzyl alcohol	C_7H_8O	108.14	Leaf	[17]
166	Phenylethyl alcohol	$C_8H_{10}O$	122.17	Leaf	[17]
167	(R)-(−)-1-Phenyl-1,2-ethanediol	$C_{10}H_{14}O_2$	166.22	Flower	[18]
168	Methyl gallate	$C_8H_8O_5$	184.15	Flower	[26]
169	Ethyl shikimate	$C_9H_{14}O_5$	202.21	Flower	[11]
170	Daucic acid	$C_7H_8O_7$	204.13	Leaf	[14]
171	Shikimic acid	$C_7H_{10}O_5$	174.15	Flower	[18]
172	(1R, 3R, 4R, 5R)-1, 3, 4, 5-tetrahydroxycyclohexanecarboxylic acid	$C_7H_{12}O_6$	192.17	Leaf	[14]
173	Stearic acid	$C_{18}H_{36}O_2$	284.48	Leaf	[27]
174	Elaidic acid	$C_{18}H_{34}O_2$	282.47	Leaf	[27]
175	Palmitic acid	$C_{16}H_{32}O_2$	256.43	Leaf	[27]
176	Dodecanoic acid	$C_{12}H_{24}O_2$	200.32	Leaf	[9]
177	β-D-glucopyranoside,3-[(1-oxo-9,12-octadecadienyl)oxy]-2-[(1-oxo-9,12,15-octadecatrienyl)oxy]propyl	$C_{44}H_{74}O_{10}$	763.07	Leaf	[2]
178	β-D-glucopyranoside,3-[(1-oxo-9,12,15-octadecadienyl)oxy]-2-[(1-oxo-9,12,15-octadecatrienyl)oxy	$C_{41}H_{66}O_{10}$	718.97	Leaf	[2]
179	β-D-glucopyranoside, 2-[[(9Z,12Z,15Z)-1-oxo-9,12,15-octadecatrien-1-yl]oxy]-3-[(1-oxooctyl)oxy]propyl	$C_{44}H_{74}O_{10}$	718.97	Leaf	[2]
180	Theobromine	$C_7H_8N_4O_2$	180.17	Leaf	[6]
181	Caffeine	$C_8H_{10}N_4O_2$	194.19	Leaf	[7]

References

1. Yang, R.; Guan, Y.; Wang, W.X.; Chen, H.J.; He, Z.C.; Jia, A.Q. Antioxidant capacity of phenolics in *Camellia nitidissima* Chi flowers and their identification by HPLC Triple TOF MS/MS. *PLoS ONE* **2018**, *13*, 20. https://doi.org/10.1371/journal.pone.0195508.
2. Zhao, M.M.; Linghu, K.G.; Xiao, L.X.; Hua, T.Y.; Zhao, G.D.; Chen, Q.L.; Xiong, S.H.; Shen, L.Y.; Yu, J.Y.; Hou, X.T.; et al. Anti-inflammatory/anti-oxidant properties and the UPLC-QTOF/MS-based metabolomics discrimination of three yellow camellia species. *Food Res. Int.* **2022**, *160*, 111628. https://doi.org/10.1016/j.foodres.2022.111628.
3. Hou, X.Y.; Du, H.Z.; Yang, R.; Qi, J.; Huang, Y.; Feng, S.Y.; Wu, Y.; Lin, S.S.; Liu, Z.X.; Jia, A.Q.; et al. The antitumor activity screening of chemical constituents from *Camellia nitidissima* Chi. *Int. J. Mol. Med.* **2018**, *41*, 2793-2801. https://doi.org/10.3892/ijmm.2018.3502.
4. Yang, R.; Wang, W.X.; Chen, H.J.; He, Z.C.; Jia, A.Q. The inhibition of advanced glycation end-products by five fractions and three main flavonoids from *Camellia nitidissima* Chi flowers. *J. Food Drug Anal.* **2018**, *26*, 252-259. https://doi.org/10.1016/j.jfda.2017.03.007.
5. Li, X.L.; Wang, J.T.; Sun, Z.Y.; Wang, J.; Yin, H.F.; Fan, Z.Q.; Li, J.Y. Flavonoid components in flowers from three species of section *Chrysantha* Chang in *Camellia*. *Guihaia* **2019**, *39*, 917-924.
6. Yao, H.Y.; Yan, G.Y.; Gao, H.; Li, Y.Y.; Bai, Y.Y.; Dong, Z.P.; Xie, Y.J. Identification of chemical constituents in the leaves of 2 species of *Camellia nitidissima* by ultra performance liquid chromatography-quadrupole-electrostatic field orbitrap high resolution mass spectrometry. *J. Food Saf. Qual.* **2022**, *13*, 8091-8100.
7. Lin, J.N.; Lin, H.Y.; Yang, N.S.; Li, Y.H.; Lee, M.R.; Chuang, C.H.; Ho, C.T.; Kuo, S.C.; Way, T.D. Chemical Constituents and Anticancer Activity of Yellow Camellias against MDA-MB-231 Human Breast Cancer Cells. *J. Agric. Food Chem.* **2013**, *61*, 9638-9644. https://doi.org/10.1021/jf4029877.
8. Peng, X.; Yu, D.Y.; Feng, B.M.; Tang, L.; Wang, Y.Q.; Shi, L.Y. Chemical constituents from the flowers of *Camellia chrysantha*. *Guihaia* **2011**, *31*, 550-553+568.
9. Qi, J.; Shi, R.F.; Yu, J.M.; Li, Y.; Yuan, S.T. Chemical Constituents from Leaves of *Camellia nitidissima* and Their Potential Cytotoxicity on SGC7901 Cells. *Chin. Herb. Med.* **2016**, *8*, 80-84.
10. Wang, Z.L.; Hou, X.Y.; Li, M.; Ji, R.S.; Li, Z.Y.; Wang, Y.Q.; Guo, Y.J.; Liu, D.H.; Huang, B.S.; Du, H.Z. Active fractions of golden-flowered tea (*Camellia nitidissima* Chi) inhibit epidermal growth factor receptor mutated non-small cell lung cancer *via* multiple pathways and targets *in vitro* and *in vivo*. *Front. Nutr.* **2022**, *9*, 1014414. https://doi.org/10.3389/fnut.2022.1014414.
11. Wang, Z.N.; Sun, B.; Yang, R.; Jia, A.Q. Flavonoids and other phenolics from *Camellia nitidissima* chi flowers. *Nat. Prod. Res.* **2023**, *37*, 180-187. https://doi.org/10.1080/14786419.2021.1960326.
12. Tian, G.H.; Wang, Z.Y.; Zhang, Y.X.; Zhao, Q.; Hou, W.B. Establishment of fingerprint, component identification, and *in vitro* antioxidant activity of *Camellia nitidissima*. *Drug*

Eval. Res. **2023**, *46*, 2387-2394.

13. Peng, X.; Yu, D.Y.; Feng, B.M.; Wang, Y.Q.; Shi, L.Y. A new acylated flavonoid glycoside from the flowers of *Camellia nitidissima* and its effect on the induction of apoptosis in human lymphoma U937 cells. *J. Asian Nat. Prod. Res.* **2012**, *14*, 799-804. https://doi.org/10.1080/10286020.2012.691475.
14. Wei, J.B.; Nong, C.L.; Su, Z.H.; Chen, Y.X.; Lv, S.J.; Pan, Y.S. Studies on Antitumor Effects of *Camellia chrysantha in vitro* and its Material Basis. *Chin. J. Exp. Tradit. Med. Formulae* **2014**, *20*, 169-174.
15. Chen, J.H.; Wu, X.H.; Zhou, Y.; He, J.H. *Camellia nitidissima* Chi leaf as pancreatic lipasen inhibitors: Inhibition potentials and mechanism. *J. Food Biochem.* **2021**, *45*, e13837. https://doi.org/10.1111/jfbc.13837.
16. Wang, B.; Ge, L.; Mo, J.G.; Su, L.; Li, Y.J.; Yang, K.D. Essential oils and ethanol extract from *Camellia nitidissima* and evaluation of their biological activity. *J. Food Sci. Technol.-Mysore* **2018**, *55*, 5075-5081. https://doi.org/10.1007/s13197-018-3446-x.
17. Huang, Y.L.; Chen, Y.Y.; Wen, Y.X.; Li, D.P.; Liu, J.L.; Wei, X. Analysis of volatile components in *Camellia nitidssima* by GC-MS. *Food Sci. Technol.* **2009**, *34*, 257-260.
18. Zhang, C.R. Chemical Constituents in Flowers from Two Camellia and Their Biological Activities. Master[illegible] thesis, Guangxi University, 2024.
19. Ge, L.; Lin, B.H.; Mo, J.G.; Chen, Q.H.; Su, L.; Li, Y.J.; Yang, K.D. Composition and antioxidant and antibacterial activities of essential oils from three yellow *Camellia* species. *Trees-Struct. Funct.* **2019**, *33*, 205-212. https://doi.org/10.1007/s00468-018-1769-x.
20. Xu, Y.A.; Ma, S.Y.; Han, X.F.; Su, L.; Ge, L.; Chen, Q.H.; Yang, K.D.; Mo, Q.F. Triterpenes and Saponins from Leaves of *Camellia nitidissima*, and Cytotoxic Activities Against Bel-7402 and SMMC-7721 Human Liver Cancer Cells. *Rec. Nat. Prod.* **2022**, *16*, 550-558. https://doi.org/10.25135/rnp.308.2111.2273.
21. Cuc, N.T.; Bang, N.A.; Tai, B.H.; Nhiem, N.X.; Yen, P.H.; Yen, D.T.H.; Hien, T.T.T.; Huyen, L.; Kiem, P.V. Five new oleanane triterpene saponins from *Camellia petelotii* and their alpha-glucosidase inhibitory activity. *Chem. Biodivers.* **2023**, *20*, e202300093. https://doi.org/10.1002/cbdv.202300093.
22. Zhao, M.; Xian, X.Y.; Yan, M.Q.; Zhou, X.L.; Huang, X.; Su, Y.Q.; Zou, D.F.; Liang, C.Q. A new oleanane-type triterpenoid saponin with a-glucosidase inhibitory activity from *Camellia nitidissima*. *J. Asian Nat. Prod. Res.* **2023**, *25*, 890-898. https://doi.org/10.1080/10286020.2022.2152012.
23. He, D.Y.; Wang, N.; Sai, X.; Li, X.Y.; Xu, Y.P. *Camellia euphlebia* protects against corticosterone-induced apoptosis in differentiated PC12 cells by regulating the mitochondrial apoptotic pathway and PKA/CREB/BDNF signaling pathway. *Food Chem. Toxicol.* **2019**, *126*, 211-222. https://doi.org/10.1016/j.fct.2019.02.028.
24. Song, L.X.; Wang, X.S.; Zheng, X.Q.; Huang, D.J. Polyphenolic antioxidant profiles of yellow camellia. *Food Chem.* **2011**, *129*, 351-357. https://doi.org/10.1016/j.foodchem.2011.04.083.
25. Tang, J.M.; Ishino, H.; Hirohashi, T.; Jiang, X.H.; Zou, R.; Wei, X.; Li, D.P.; Ishimaru, K. Analysis of volatile components in flowers of five Sect. Chrysantha species by GC-MS. *Przem. Chem.* **2023**, *102*, 650-656. https://doi.org/10.15199/62.2023.7.2.

26. Jiang, H.; Wang, Z.N.; Jia, A.Q. Methyl gallate from *Camellia nitidissima* Chi flowers reduces quorum sensing related virulence and biofilm formation against *Aeromonas hydrophila*. *Biofouling* **2024**, *40*, 64-75. https://doi.org/10.1080/08927014.2024.2316611.
27. Wei, Q.; Zhang, L.Y. Comparative Analysis of Aroma Compositions of Two *Camellia Chrysantha* (Hu) Tuyama Species. *Mod. Food Sci. Technol.* **2013**, *29*, 668-672.
